# Apical surface supracellular mechanical properties in polarized epithelium using noninvasive acoustic force spectroscopy

**DOI:** 10.1038/s41467-017-01145-8

**Published:** 2017-10-18

**Authors:** Alexander X. Cartagena-Rivera, Christina M. Van Itallie, James M. Anderson, Richard S. Chadwick

**Affiliations:** 1 Section on Auditory Mechanics, National Institute on Deafness and Other Communication Disorders, National Institutes of Health, Bethesda, MD 20892 USA; 2 Laboratory of Tight Junction Structure and Function, National Heart, Lung, and Blood Institute, National Institutes of Health, Bethesda, MD 20892 USA

## Abstract

Maintenance of epithelial tissue integrity requires coordination between cell–cell adherens junctions, tight junctions (TJ), and the perijunctional actomyosin cytoskeleton. Here we addressed the hypothesis that alterations in TJ structure and remodeling of the actomyosin cytoskeleton modify epithelial mechanics. Current methods to measure supracellular mechanical properties disrupt intact monolayers, therefore, we developed a novel method using noncontact acoustic frequency-modulation atomic force microscopy (FM-AFM) and tested it on MDCK polarized monolayers. Our results show that double knockdown (dKD) of ZO-1/ZO-2 elevates the apical epithelial tension and effective viscosity. Interestingly, epithelial tension is more sensitive to inhibition of myosin II ATPase activity than to inhibition of ROCK activity, but viscosity is highly sensitive to both. Additionally, we showed epithelial intercellular pulling forces at tricellular junctions and adhesion forces in dKD cells are elevated with an increase in contractility. In conclusion, FM-AFM enables the physiological and quantitative investigation of mechanics in intact epithelium.

## Abstract (web summary)

Determination of apical tension, fluidity, and intercellular adhesive forces in an epithelial monolayer are currently disruptive. Here the authors present a method using acoustic force microscopy to measure changes in these parameters upon tight junction structural alterations in a MDCK monolayer.

## Introduction

Adhesive forces are crucial for maintaining epithelial tissue integrity. These forces must be dynamic to maintain stable cell associations under external stress by complex processes such as cell movements and epithelial morphogenesis. The structures that form and maintain cell–cell contacts include adherens junctions (AJ)^1^, tight junctions (TJ)^2^, desmosomes^3^, and gap junctions^4^. The adherens and tight junctions are continuous adhesive contacts adjacent to the apical cell surface, while desmosomes and gap junctions form punctate contacts along the lateral cell–cell contacts. Thus, the AJ and TJ are assumed to be the main contributors to apical surface mechanical properties in polarized epithelium.

**Fig. 1:**
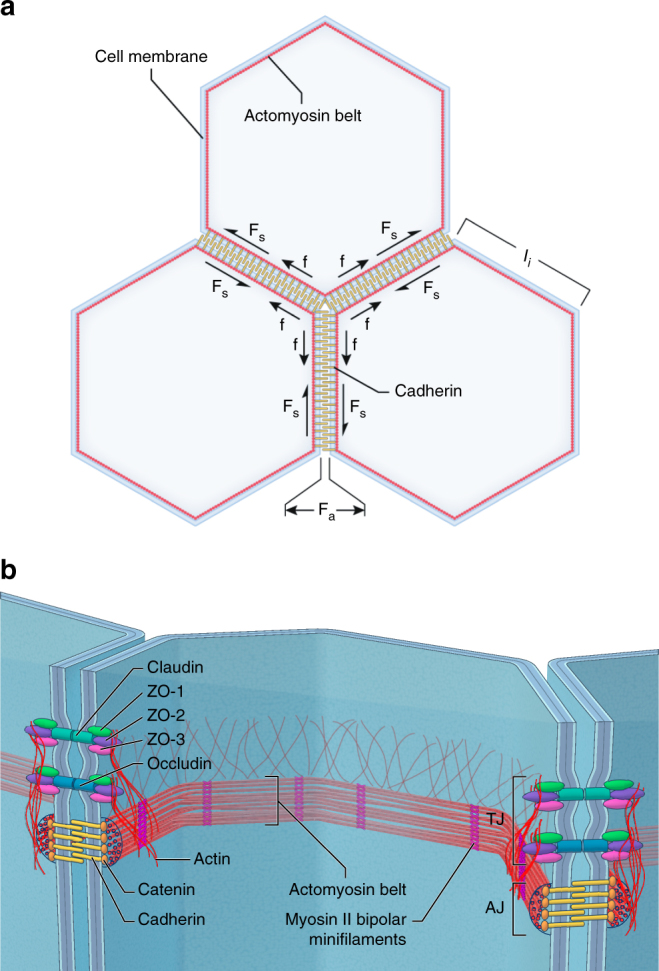
Critical intra- and intercellular forces acting within a polarized epithelium and key molecular structures. **a** The illustration shows the working model for polarized epithelium tension and intercellular adhesion forces. Cell–cell contacts transmit mechanical tension through a dynamic coupling between adherens and tight junctions and the perijunctional actomyosin cytoskeleton. The epithelium contractility generates an isotropic tension balanced by the intercellular normal and shear adhesive forces. The free body diagram presents the variables used in our theoretical model where; *f* is the pulling force acting at tricellular junctions, *F*_NA_ is the intercellular normal adhesive force, *F*_SA_ is the intercellular shear adhesive force, and *l*_*i*_ is the cell edge length. **b** Diagram illustrating the localization and molecular structural composition of the actomyosin cytoskeleton belt interacting with tight and adherens junctions. In the tight junctions the ZO-1, ZO-2, and ZO-3 proteins bind to the transmembrane receptor proteins claudins and occludins and to the perijunctional actin cytoskeleton plaque. In the adherens junctions catenin proteins bind to the transmembrane cadherins and to the actomyosin belt with large F-actin bundles and bipolar myosin II bipolar minifilaments motors

Intercellular force transmission is normally attributed to AJs, because they are known to create a physical connection between the perijunctional actomyosin cytoskeleton just inside the lateral plasma membrane near the apical junctions and intercellular adhesion molecules of the cadherin superfamily^5^, (Fig. 1). For example, in Madin-Darby canine kidney (MDCK) polarized monolayers, the classical epithelial cadherin (E-cadherin) is one of the critical proteins responsible for force transmission and strength of the epithelium^6^. Moreover, it is thought that AJ tension-sensitive regulation of the actomyosin cytoskeleton may help reinforce cell–cell adhesions against increased intercellular forces in epithelial monolayers^7, 8^. Besides the AJs, the actomyosin cytoskeleton of adjacent epithelial cells is also connected to the transmembrane adhesion proteins of the TJs^9^. Tight junctions are commonly associated with transepithelial barrier formation rather than force transmission^10^, but a contribution to force transmission and epithelial mechanical strength has not been ruled out.

The TJ scaffolding proteins, including ZO-1, ZO-2, and ZO-3, are intracellular linkers that directly connect the actomyosin cytoskeleton with transmembrane adhesion proteins, including claudins, occludin, and JAM-A^9^, (Fig. 1). The depletion of ZO-1 and/or ZO-2 has been shown to cause dramatic reorganization of the perijunctional actomyosin cytoskeleton into large thick arrays of F-actin under the adherens junction contacts and recruitment of myosin II bipolar minifilaments, forming a contractile sarcomeric belt-like distribution^11^. Using laser ablation to cut cell–cell contacts, it was shown that elevated junctional contractility in ZO KD cells results in a dramatic increase in recoil velocity, implying an elevated epithelial tension^12^. However, laser ablation is a disruptive technique, and cutting the cell–cell contacts of adjacent cells makes it difficult to determine any specific contribution of alterations of tight junction structure to epithelial tension and intercellular forces.

The organization of filamentous actin and myosin II bipolar minifilaments is critical for defining the mechanical properties of individual cells and multicellular tissues, and thus plays an important role in cellular processes such as migration and morphogenesis^13^. For example, the highly contractile actomyosin cortex in melanoma cells under confinement was shown to create elevated cortical tension and intracellular pressure and thus be the main factor that drives large bleb formation and unregulated amoeboid migration^14^. In epithelial monolayers extruding apoptotic cells, it was shown that coronin 1B is recruited to E-cadherin contacts reorganizing the actomyosin cytoskeleton at the apoptotic cell interface, and elevating the tension for effective apoptotic cell clearance^15^. Therefore, quantitative determination of critical physical parameters is key for understanding how forces are transmitted within and between cells during important cellular processes and for providing insight into regulation of these processes.

Atomic force microscopy (AFM) is a biophysical technique that does not require invasive disruption nor chemical coupling between the probe and the biological sample. AFM is capable of providing topographical and mechanical property measurements of cells and tissues in their native physiological environments at high spatiotemporal resolution (a few seconds per 256 by 256 pixels/frame with sub-100 nm spatial resolution)^16^. Recently, using AFM quasi-static force-distance curves with tipless microcantilevers, it was shown that cortical tension, elastic Young’s modulus, and intracellular pressure in nonadherent cells can be measured by molecular perturbations to the actomyosin cortex^17^. This work showed that myosin II ATPase activity and unbranched F-actin polymerization increase cortex tension and intracellular pressure, while branched actin networks decrease them^17^. Moreover, AFM force curves using cantilevers with attached microbeads were used to dissect the cortical tension and stiffness of germ-layer progenitors in zebrafish, indicating that cortical mechanical properties govern the germ-layer organization^18^. Membrane tension of individual epithelial cells within confluent monolayers has been measured by AFM tether-pulling and nanoindentations, yielding important insight about how mechanical properties contribute to tissue osmoregulation^19, 20^. Although these AFM methodologies have been developed to measure the elastic mechanical properties of single cells, they are unable to measure epithelial tension, viscosity, and intercellular forces in multicellular systems.

Here we modified a recently described noncontact frequency modulation atomic force microscopy (FM-AFM) method^21^ to determine apical surface epithelial tension, effective viscosity, and intercellular adhesive forces. Noninvasive FM-AFM is based on the phenomenon that when a cantilever with an attached micron-sized bead is acoustically vibrating in a liquid environment and approaches from a few microns to within hundreds of nanometers from the sample surface, a hydrodynamic interaction will cause a frequency shift to the cantilever that can be easily measured with the sensitive AFM detection system^21–23^. We developed a theoretical mathematical model based on lubrication theory for linearized unsteady Stokes flow, and applied it to a micron-sized sphere vibrated at acoustic frequencies with small oscillation amplitude as it approaches a compliant substrate. We combined the FM-AFM frequency shift measurements with the new theoretical model to better understand how ZO proteins regulate the apical epithelial tension, effective viscosity, and intra–intercellular forces in polarized monolayers of MDCK II cell lines. This novel method has the ability to measure the supracellular mechanical properties of polarized epithelia limited to the very apical plane (maximum depth ~ 2.5 µm), which is essential to investigate the mechanics of the apical junction complex (tight and adherens junctions) and minimize contributions from middle (desmosomes and gap junctions) and basal (focal adhesions) planes.

Using Tet-off MDCK II cell lines in which both ZO-1 and ZO-2 were depleted, we observed a dramatic increase in apical epithelial tension and effective viscosity in these double knockdown cells. This is consistent with the observation of a striking thickening of contractile actomyosin arrays associated with the AJ in ZO-deficient monolayers^11^. Moreover, we observed that myosin II ATPase activity is the major contributor to elevated tension, since inhibition of ATPase activity with blebbistatin, but not inhibition of Rho-kinase (ROCK) activity with Y-27632 decreased epithelial tension. In contrast, we observed that the maintenance of normal tissue fluidity was sensitive to treatment with both blebbistatin and the ROCK inhibitior. Additionally, a simple force balance involving two connected tricellular junctions permits the calculation of the intercellular adhesion forces (normal and shear adhesive forces) from the measured epithelial tension, the length of cell edges, and angular geometry. These results suggest that intercellular normal and shear adhesive forces are critical for maintenance of tissue stability and avoiding rupture of the adhesive contacts. Altogether, the study shows that this simple noninvasive FM-AFM method can be used to investigate the biomechanical feedback on tissues and could provide important insights into the molecular regulation of epithelial organization and morphogenesis.

## Results

### Polarized epithelial tension theory

We develop a new mathematical theory to determine the epithelial tension in mature polarized monolayers having a polygonal cellular pattern and mature cell–cell junctions. Mature cell–cell adhesions have fully developed adherens junctions, with transmembrane cadherin proteins linked through catenins to an ultrastructurally distinct highly contractile actomyosin belt and tight junctions, with transmembrane adhesion proteins claudins, occludin, and JAM-As linked through ZO proteins to a less distinct actin cytoskeleton plaque (Fig. 1b). Notably, it is poorly understood whether these two distinct actin cytoskeleton structures are linked and if so how. Contractile activity of actomyosin creates a cortical tension that is transmitted to the cell–cell junctions. This generates tension in the epithelium apical surface that is balanced by intercellular forces protecting the integrity of the epithelium (Fig. 1a).

**Fig. 2:**
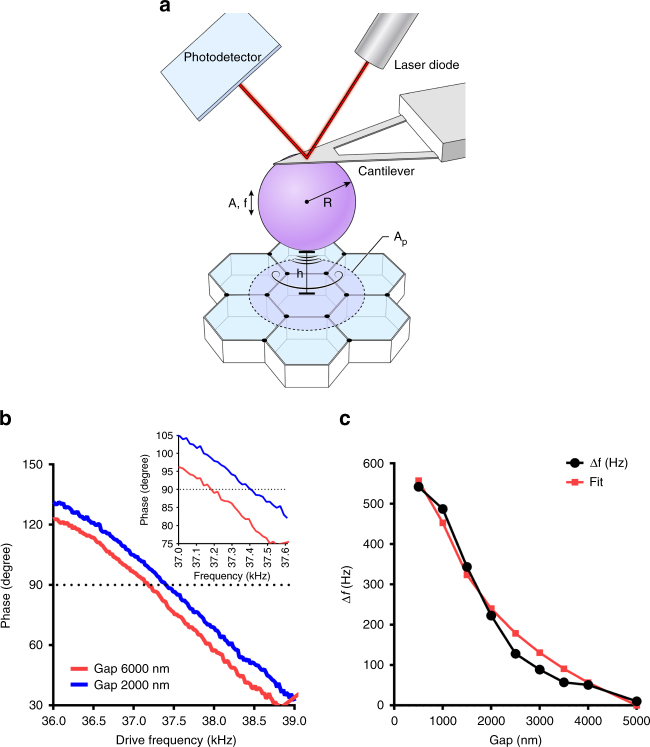
Measurement of frequency shifts to determine apical epithelium mechanical properties. **a** Schematic diagram of the noncontact acoustic frequency modulation method to measure epithelial apical surface mechanical properties. As the acoustically vibrating cantilever with a microsphere approaches the epithelium, a hydrodynamic interaction emerges and increases monotonically as the sphere gets closer. This interaction can be measured by the AFM as an increase in frequency shift. *f* is the cantilever drive frequency, *∆f* is the frequency shift, *A* is the oscillation amplitude, *R* is the microsphere radius, *h* is the distance between the microsphere and the epithelium apical surface, and *A*_p_ is the effective probed area. **b** Phase-frequency response curves showing frequency shifts acquired by keeping *π*/2 phase when the acoustically vibrating microsphere is moved closer to the apical epithelium surface. Dotted line represents *π*/2 phase. **c** Representative example of frequency shift measurements performed on a MDCK II polarized monolayer at different distances. Reconstructed frequency shift-gap curves were then fitted using Eq. (1) to determine the apical epithelial surface tension. The red line depicts the fitted curve

Noninvasive acoustic FM-AFM is based on a micron-sized rigid sphere attached to the end of a AFM microcantilever with calibrated spring constant oscillating at acoustic frequencies (kHz) with nanometer oscillation amplitudes (typically 10 nm) in an incompressible fluid bath (Fig. 2a). When the acoustically oscillating sphere approaches a compliant epithelium, the material properties of the epithelium can be measured through changes in cantilever resonant frequency based on the hydrodynamics of thin gap interactions between the sphere and substrate^21^. The sphere oscillation amplitude is very small compared to the minimum gap height, which in turn is small compared to the sphere radius.

Our theoretical model to calculate the apical surface epithelial tension in a polarized epithelium from cell–cell adhesion and steady-state active prestress caused by myosin II activity using frequency shifts requires some critical assumptions. An important assumption is that a polarized confluent sheet of fully developed cells produce an isotropic epithelial tension in its apical surface. The epithelial tension generated by an array of polygonal cells whose boundaries are under line tension is isotropic under some conditions (Supplementary Note 1). Additionally, we ignored epithelial viscoelastic effects, because the magnitude of effective viscosity of the polarized monolayer shows the tension measurement is affected by <10% (Supplementary Note 3). We also assume that the presence of microvilli does not considerably affect the epithelial tension. A recent study showed that AFM can be used to detect the epithelial apical surface microvilli (brush-like layer) when contacting the apical surface of individual normal and cancerous epithelial cells; however, the extracted elastic Young’s modulus values were not statistically significant^24^. Development of such a full-scale model considering the viscous dissipation and small effect of microvilli to apical surface epithelial mechanics is unnecessary for the present work. The Supplementary Note 1 contain a full description of the theoretical model derivation of epithelial tension on a compliant and confluent monolayer. The final formula describing the relationship between frequency shift and epithelial tension is:

1 $$\Delta f = \frac{4}{{{k_c}R}}\sqrt {\frac{{\pi {f_{{\rm{far}}}}{h_{\rm{m}}}{T^3}}}{{6\mu }}} \left( {\frac{{{h_{{\rm{far}}}}}}{{{h_{\rm{m}}}}} - 1} \right),$$

where ∆*f*=*f*_near_−*f*_far_ (Hz) is the frequency shift where the phase of the piezo to that of the microsphere is *π*/2 (Fig. 2b), *f*_far_ (Hz) is the “unperturbed” cantilever resonance frequency far away from the sample surface where the phase is *π*/2, *f*_near_ (Hz) is the “perturbed” cantilever resonance frequency near the sample surface where the phase is *π*/2, *k*_c_ (N m^−1^) is the cantilever spring constant, *R* (m) is the radius of the cantilever microsphere, *h*_far_ (m) is the farthest distance between the lowest portion of the sphere and the epithelial apical surface where the cantilever dynamics are unperturbed, *h*_m_ (m) is the minimum distance between the lowest portion of the sphere and the epithelial surface as the bead is moved closer, *µ* (Pa-s) is the incompressible fluid viscosity, and *T* (N m^−1^) is the epithelial tension.

To measure epithelial tension, we moved the acoustically vibrating sphere closer to the apical surface of the MDCK II polarized epithelium and recorded each frequency sweep from 6 µm to 500 nm with 500 nm intervals. During experiments, we recorded frequency sweeps at different distances around the resonance frequency *f*_*π*/2_, where the phase was set to *π*/2 at a distance of 6 µm (Fig. 2b). In Fig. 2b, it can be observed that as the vibrating cantilever with a 25 µm bead was moved closer from 6 to 2 µm to a confluent MDCK II epithelium, the frequency *f*_*π/2*_ shifted to higher frequencies. Figure 2c is a representative frequency-gap curve and shows the monotonically increase in frequency shift due to increase in the strength of the hydrodynamic interaction forces as the vibrating microbead is moved closer to the epithelium apical surface. Equation 1 is used to fit the data and calculate the epithelial tension. The red line represents the data fit (generated using nonlinear least squares method to best fit the data). The excellent overlapping agreement indicates that the theoretical model fits very well the experimental data.

Since a polarized epithelium is a complex three-dimensional biological soft material, we tested how reasonable our described method is to measure the epithelial tension large-scale mechanical property. We used two additional micron size beads (20 µm and 35 µm) to assess the quantitative robustness of the described method. First, we computed the effective probe diameter (${D_{{\rm{eff}}}} = 4{\left( {R{h_{\rm{m}}}} \right)^{1{\rm{/}}2}}$) and observed that for distances 500 nm to 1 µm onwards from the epithelium surface the probes measure material properties larger than a single cell (typically 10 µm) and is therefore not a very localized measurement, (Supplementary Fig. 3). Then, using Eq. (1) to measure the apical surface epithelial tension in MDCK II polarized monolayers for all the bead sizes we observed no significant differences between them, (Supplementary Fig. 4). The fact that the extracted epithelial tension in the monolayers using additional bead sizes (20 µm and 35 µm) compared very well with obtained tension values using 25 µm supports the quantitative robustness of the described method.

### Epithelial tension increases in ZO-depleted MDCK monolayers

**Fig. 3:**
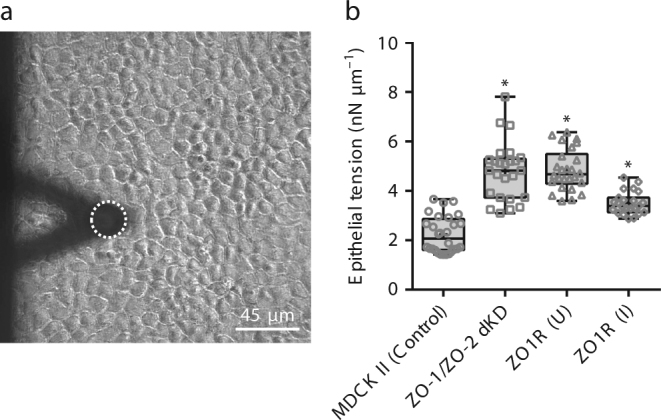
Alterations of tight junctions structure by depletion of ZO-1 and ZO-2 elevates polarized epithelial tension. **a** Phase contrast image showing the AFM cantilever with microbead (dashed circle) and the MDCK II polarized monolayer. **b** In ZO-1/ZO-2 dKD in MDCK II Tet-off cells the apical surface epithelial tension dramatically increases. In ZO1R(U) dKD cells the apical surface epithelial tension is significantly elevated when compared to control, but the tension is partially restored when ZO1R(I) dKD cells are expressing a Tet-inducible full-length ZO-1 rescue transgene. U, ( + dox, uninduced); I, (−dox, induced). Data are represented as mean ± s.d.; * indicates significant difference in comparison with control *P* < 0.05 (unpaired two-tailed Student’s *t*-test with Welch’s correction); number of measurements pooled for 2–3 independent experiments for each condition, *n* = 28, 25, 26, and 23 for control, ZO-1/ZO-2 dKD, ZO1R(U), and ZO1R(I), respectively

We next sought to test the hypothesis that depletion of the tight junction proteins ZO-1 and ZO-2 causes a significant effect on the epithelial tension on the apical surface of MDCK II monolayers. We measured epithelial tension in MDCK II (control), a double knockdown of ZO-1 and ZO-2 (ZO-1/ZO-2 dKD), and an inducible rescue with ZO-1 (ZO1R (U; uninduced) and ZO1R (I; induced)) cell lines by performing noncontact FM-AFM experiments after culturing the cells for 7–10 days to form a polarized epithelium (Fig. 3a). Measured epithelial tensions were: control *T*=2.2 ± 0.7 nN µm^−1^ (mean ± s.d.), ZO-1/ZO-2 dKD *T* = 4.8 ± 1.2 nN µm^−1^, ZO1R(U) *T* = 4.9 ± 0.8 nN µm^−1^, and ZO1R(I) *T* = 3.5 ± 0.5 nN µm^−1^. Compared to the control MDCK II cell line, three separate double knockdown clones demonstrated significant differences in measured tension with ~ 55% increase seen for ZO-1/ZO-2 dKD and ZO1R(U), while only ~ 30% increase for ZO1R(I) monolayers (Fig. 3b). These results show that removing the cytoskeletal linkers ZO-1 and ZO-2 dramatically increases the epithelial tension. Interestingly, it appears that both ZO-1 and ZO-2 are required to achieve normal epithelial tension, as re-expression of full-length ZO-1 in the ZO1R cell line results only in a partial rescue. Altogether, the results demonstrate the sensitivity of the method by measuring changes in epithelial tension with a partial phenotype rescue.

We also measured the apical surface epithelial elastic Young’s modulus in our MDCK II cell lines, using standard nanoindentation force spectroscopy. Similar to epithelial tension, the elastic Young’s modulus was also elevated in ZO-depleted MDCK II polarized epithelium; however, the relative differences are less dramatic (Supplementary Fig. 5). Altogether, these results show that our noninvasive FM-AFM method provides an increase in the sensitivity for measuring epithelium mechanics compared with commonly used force curves.

### Actomyosin belt contractility regulates epithelial tension

**Fig. 4:**
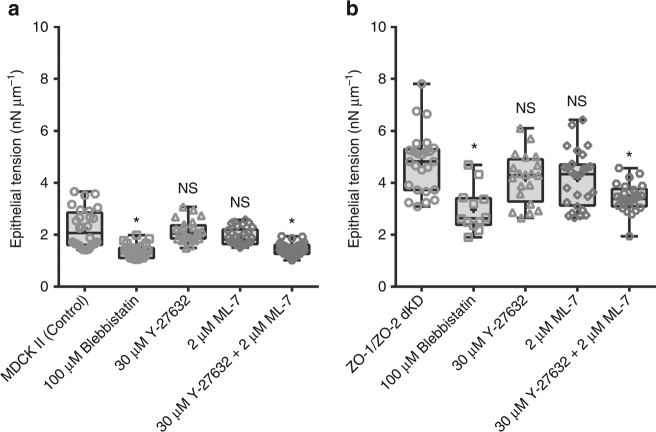
Apical surface tension in polarized epithelium is mostly determined by myosin II motor ATPase activity rather than ROCK signaling or MLCK activity. Confluent monolayers of MDCK II controls and ZO-1/ZO-2 dKD cells were treated with 100 µM blebbistatin, 30 µM Y-27632, 2 µM ML-7, or 30 µM Y-27632 + 2 µM ML-7 pharmacological drugs for 15 to 20 h. **a** Epithelial tension of MDCK II control untreated polarized monolayers, and treated either with 100 µM blebbistatin, 30 µM Y-27632, 2 µM ML-7, or 30 µM Y-27632 + 2 µM ML-7. **b** Epithelial tension of MDCK II ZO-1/ZO-2 dKD untreated polarized monolayers, or treated either with 100 µM blebbistatin, 30 µM Y-27632, 2 µM ML-7, or 30 µM Y-27632 + 2 µM ML-7. Myosin II activity inhibition significantly reduces the epithelial tension, whereas individual inhibition of ROCK signaling or MLCK activity do not cause a significant change on epithelial tension. Interestingly, combined inhibition of ROCK signaling and MLCK activity significantly reduces the epithelial tension. Data are represented as mean ± s.d.; * indicates significant difference in comparison with control *P* < 0.05 (unpaired two-tailed Student’s *t*-test with Welch’s correction); *NS* nonsignificant differences in comparison with control *P* > 0.05(unpaired two-tailed Student’s *t*-test with Welch’s correction); number of measurements pooled for 2–3 independent experiments for each condition, *n* = 28, 27, 24, 30, and 29 for control, 100 µM blebbistatin, 30 µM Y-27632, 2 µM ML-7, and 30 µM Y-27632 + 2 µM ML-7, respectively, and *n* = 25, 15, 20, 28, and 28 for ZO-1/ZO-2 dKD, 100 µM blebbistatin, 30 µM Y-27632, 2 µM ML-7, and 30 µM Y-27632 + 2 µM ML-7, respectively

We next tested the contribution of myosin II motor activity to the elevated epithelial tension observed in dKD monolayers. We treated monolayers for 15 to 20 h with 100 µM blebbistatin to inhibit myosin II motor ATPase activity^25^, with 30 µM Y-27632 to inhibit ROCK signaling upstream myosin activation^26^, or with 2 µM ML-7 to inhibit myosin light chain kinase (MLCK) activity^27^. Epithelial tension for the MDCK II control monolayers was *T* = 2.2 ± 0.7 nN µm^−1^, and for the monolayer treated with either 100 µM blebbistatin *T* = 1.4 ± 0.3 nN µm^−1^, 30 µM Y-27632 *T* = 2.1 ± 0.4 nN µm^−1^, 2 µM ML-7 *T* = 1.9 ± 0.3 nN µm^−1^, or 30 µM Y-27632 + 2 µM ML-7 *T* = 1.5 ± 0.2 nN µm^−1^, (Fig. 4a). The measured epithelial tensions for the ZO-1/ZO-2 dKD monolayers were: control *T* = 4.8 ± 1.2 nN µm^−1^, and treated with either 100 µM blebbistatin *T* = 3 ± 0.9 nN µm^−1^, 30 µM Y-27632 *T* = 4.2 ± 1 nN µm^−1^, 2 µM ML-7 *T* = 4.2 ± 1.1 nN µm^−1^, or 30 µM Y-27632 + 2 µM ML-7 *T* = 3.4 ± 0.5 nN µm^−1^ (Fig. 4b). As expected and consistent with other studies^11, 12^, inhibition of myosin II ATPase activity by blebbistatin significantly reduced the epithelial tension on control and dKD monolayers. Interestingly, control and dKD confluent monolayers were strikingly insensitive to individual inhibition of ROCK signaling with Y-27632 or MLCK activity with ML-7, both upstream of myosin II activation. However, it has been reported that Y27632 and ML-7 do not have equivalent effects on all phosphorylation states of MLCK^27^ and we did find that simultaneous treatment with Y-27632 and ML-7 significantly reduced epithelial tension (Fig. 4), suggesting regulation of myosin II activity is complex and regulated by multiple upstream pathways. Additionally, we measured the epithelial elastic Young’s modulus by nanoindentation experiments and obtained the same results with blebbistatin reducing the epithelium stiffness, whereas treatment with Y-27632 or ML-7 did not result in noticeable changes (Supplementary Fig. 6). Altogether, these results suggest that myosin’s II ATPase activity on polarized monolayers is critical to epithelial integrity.

### Apical effective viscosity is elevated in dKD monolayers

We extended our approach to estimate the epithelial fluidity through the effective viscosity of apical surface polarized epithelium. There is mounting evidence suggesting that tissues are viscoelastic and viscous dissipation plays a significant role in their surface mechanical properties^28, 29^. We treated a confluent monolayer as a compliant homogeneous substrate prestressed by an isotropic tension *T* to obtain an analytical equation to measure the tissue effective viscosity. See Supplementary Note 1 for detailed derivation. The effective viscosity is determined from:

2 $${\mu _{{\rm{eff}}}} = - \frac{{12T}}{R}\left( {\frac{\lambda }{{{h_{\rm{m}}}}}} \right)\frac{1}{{\pi {f_{{\rm{far}}}^2}\frac{{d\phi }}{{df}}}},$$

where *µ*_eff_ is the substrate effective viscosity (Pa-s), $\lambda = 1{\rm{/}}2{\left( {R{h_m}} \right)^{1{\rm{/}}2}}$ is the penetration depth, *ϕ* is the cantilever response phase, and $\frac{{{\rm{d}}\phi }}{{{\rm{d}}f}}$ the slope of the phase-frequency curve.

**Fig. 5:**
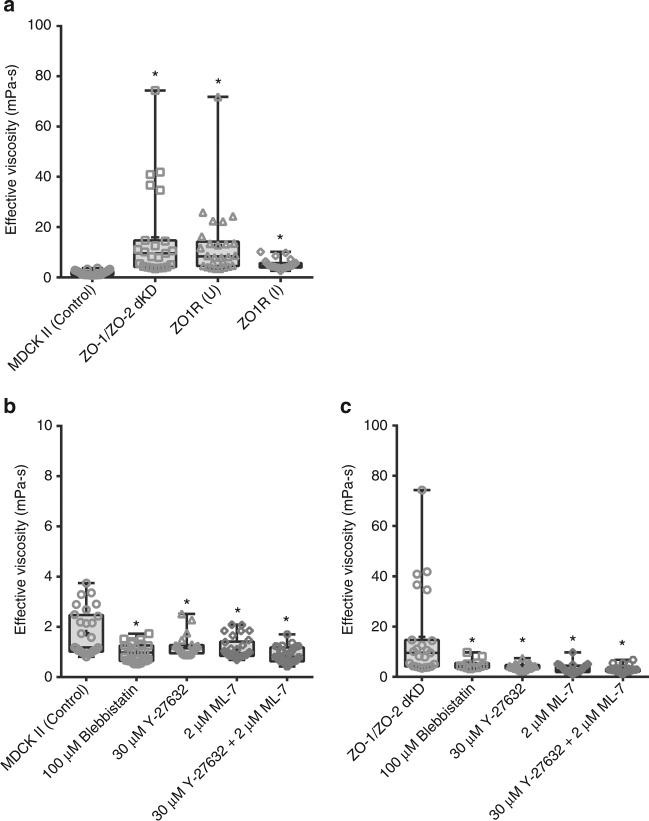
Apical surface effective viscosity is elevated in ZO-1/ZO-2 dKD polarized epithelium and additional actomyosin perturbations significantly modify its behavior. **a** ZO-1/ZO-2 dKD in MDCK II Tet-off cells significantly increases the epithelial effective viscosity. Number of measurements pooled for 2–3 independent experiments for each condition, *n* = 28, 25, 26, and 23 for control, ZO-1/ZO-2 dKD, ZO1R(U), and ZO1R(I), respectively. **b** Epithelial effective viscosity of MDCK II control untreated polarized monolayers, and treated either with 100 µM blebbistatin, 30 µM Y-27632, 2 µM ML-7, or 30 µM Y-27632 + 2 µM ML-7. **c** Epithelial effective viscosity of MDCK II ZO-1/ZO-2 dKD untreated polarized monolayers, or treated either with 100 µM blebbistatin, 30 µM Y-27632, 2 µM ML-7, or 30 µM Y-27632 + 2 µM ML-7. Inhibition of myosin II ATPase activity, ROCK signaling, and MLCK activity significantly reduces the epithelial effective viscosity. Combine inhibition of ROCK signaling and MLCK activity further reduces the epithelial viscosity. Confluent monolayers of MDCK II controls and ZO-1/ZO-2 dKD cells were treated with the pharmacological drugs for 15 to 20 h. Number of measurements pooled for 2–3 independent experiments for each condition, *n* = 28, 27, 24, 30, and 29 for control, 100 µM blebbistatin, 30 µM Y-27632, 2 µM ML-7, and 30 µM Y-27632 + 2 µM ML-7, respectively, and *n* = 25, 15, 20, 28, and 28 for ZO-1/ZO-2 dKD, 100 µM blebbistatin, 30 µM Y-27632, 2 µM ML-7, and 30 µM Y-27632 + 2 µM ML-7, respectively. All data are represented as mean ± s.d.; * indicates significant difference in comparison with control *P* < 0.05 (unpaired two-tailed Student’s *t*-test with Welch’s correction)

We then examined the apical surface effective viscosity changes in polarized epithelium with alterations in tight junctions. We measured epithelial effective viscosity in MDCK II (control), ZO-1/ZO-2 dKD, and inducible rescue with ZO-1 [ZO1R(U) and ZO1R(I)] cell lines, (Fig. 5a). Measured epithelial effective viscosities were: control *µ*_eff_=1.8 ± 0.9 mPa-s, ZO-1/ZO-2 dKD *µ*_eff_ = 15.9 ± 17.8 mPa-s, ZO1R(U) *µ*_eff_ = 12.9 ± 13.8 mPa-s, and ZO1R(I) *µ*_eff_ = 5.3 ± 1.9 mPa-s. Compared to the control MDCK II cell line, three separate double knockdown clones demonstrated significant differences in measured effective viscosity with seven- to ninefold increase was seen for ZO-1/ZO-2 dKD and ZO1R(U), while threefold increase for ZO1R(I) monolayers (Fig. 5a). These results indicated, consistent with the increase in epithelial tension, that cytoskeletal linkers ZO-1 and ZO-2 are essential for normal tissue fluidity.

### Actomyosin belt alterations modify tissue viscosity

We next focused on understanding the tissue fluidity changes with actomyosin dynamic alterations. The effective viscosity for MDCK II control monolayers was *µ*_eff_ = 1.8 ± 0.9 mPa-s, and for the monolayer treated with either 100 µM blebbistatin *µ*_eff_ = 1 ± 0.4 mPa-s, 30 µM Y-27632 *µ*_eff_ = 1.2 ± 0.4 mPa-s, 2 µM ML-7 *µ*_eff_ = 1.1 ± 0.4 mPa-s, or 30 µM Y-27632 + 2 µM ML-7 *µ*_eff_ = 0.9 ± 0.3 mPa-s, (Fig. 5b). Additionally, the measured effective viscosity for the ZO-1/ZO-2 dKD monolayers were: control *µ*_eff_ = 15.9 ± 17.8 mPa-s, and treated with either 100 µM blebbistatin *µ*_eff_ = 5 ± 2 mPa-s, 30 µM Y-27632 *µ*_eff_ = 4 ± 1.3 mPa-s, 2 µM ML-7 *µ*_eff_ = 3.3 ± 1.8 mPa-s, or 30 µM Y-27632 + 2 µM ML-7 *µ*_eff_ = 2.9 ± 1.3 mPa-s, (Fig. 5c). We observed that the monolayer effective viscosity is greatly reduced for all actomyosin perturbations. In contrast with the epithelial tension results, myosin II ATPase activity and myosin activation by ROCK or MLCK signaling activity are critical for normal tissue fluidity.

### Increased pulling forces at tricellular junctions in dKD

To calculate the intercellular pulling forces acting at tricellular junctions on a confluent epithelium, consider a sheet of confluent cells comprised of hexagons with cell edges *l*, as shown in Fig. 1a. We assumed that myosin II bipolar minifilaments exert an intercellular pulling force (*f*) away from each tricellular junction because myosin II contracts along the hexagon lateral walls (actomyosin belt). The pulling force acting on the tricellular junctions is different than the isotropic epithelial tension (*T*); however, it can be determined from the measured epithelial tension by (Supplementary Note 2 for detailed derivation and Supplementary Fig. 2):

3 $$f = \sqrt 3 Tl.$$

**Fig. 6:**
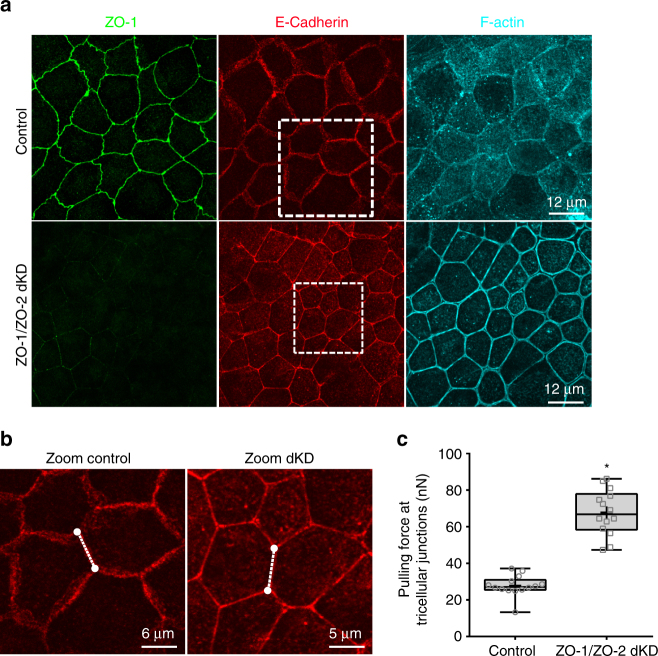
Pulling forces at tricellular junctions increases with depletion of ZO-1 and ZO-2. **a** Representative confocal images of polarized monolayers of MDCK II Tet-Off controls and ZO-1/ZO-2 dKD cells were grown on glass dishes, fixed, and stained with Alexa Fluor 647-phalloidin (F-actin) and antibodies against tight junction protein ZO-1, the adheren junction protein E-cadherin. The cell shapes of dKD cells are different (more polygonal) than controls, and the adherens cell–cell contacts are straighter. Boxed areas are shown zoomed in (**b**). (**b**) Zoomed areas of E-cadherin images depict pair of cells with hexagonal shapes within the polarized epithelium. Dashed lines represent the cell–cell junctions utilized for length estimation. All confocal images are 1.05 µm thick maximum intensity Z-projections. **c** The plot show that for ZO-1/ZO-2 dKD monolayers the pulling forces acting at tricellular junctions significantly increases compared to controls (*F*_control_ = 27.8 ± 5.7 nN vs. *F*_dKD_ = 67.8 ± 12.4 nN). In **c**, data are represented as mean ± s.d.; * indicates significant difference in comparison with control *P* < 0.05 (unpaired two-tailed Student’s *t*-test with Welch’s correction); number of measurements pooled for each condition *n* = 14

To determine the intercellular pulling forces at tricellular junctions, we needed to measure the cell–cell junction length. To do this we fixed MDCK II monolayers cultured for 7–10 days, labeled them for ZO-1 for TJ, E-cadherin for AJ, and with Alexa Fluor 647-phalloidin for F-actin, and acquired images using confocal fluorescence microscopy. In Fig. 6a maximum intensity Z-projections are shown of ZO-1, E-cadherin, and F-actin in MDCK II controls and ZO-1/ZO-2 dKD polarized monolayers at a depth of 1.05 µm. When the TJ structure was altered by depletion of ZO-1 and ZO-2, a significant change in cell shape and morphology was observed with a more polygonal shape and straighter cell–cell junctions. In addition, a striking change in F-actin distribution was noted with an actin staining enrichment at the cell–cell junctions. Because AJs are known to be the major contributor to force transmission, and they are found in both control and ZO-1/ZO-2 dKD monolayers, we chose to use E-cadherin immunofluorescence images to measure cell–cell junction lengths. We extracted the intensity vs. distance profiles for lines that were drawn parallel to individual cell–cell junctions with both cells exhibiting hexagonal shape (Fig. 6b). The lengths of intensity curves for E-cadherin fluorescence were determined by measuring the distance between the vertices on the intensity vs. distance profiles.

Depletion of ZO-1 and ZO-2 leads to significant increase in pulling forces at tricellular junctions in MDCK II monolayers. After measuring the cell–cell junction lengths from E-cadherin immunofluorescence images, we used Eq. (3) to determine the pulling force at tricellular junctions. We observed a significant increase of ~ 60% in pulling force on ZO-1/ZO-2 dKD polarized monolayers (Fig. 6c). Together the data show that increased apical surface epithelial tension on MDCK II polarized monolayers is transmitted directly to the tricellular junctions, as in ZO-1/ZO-2 dKD cells the pulling forces at tricellular junctions are dramatically increased and the cell–cell junctions are straighter.

### The intercellular adhesive forces in polarized monolayers

**Fig. 7:**
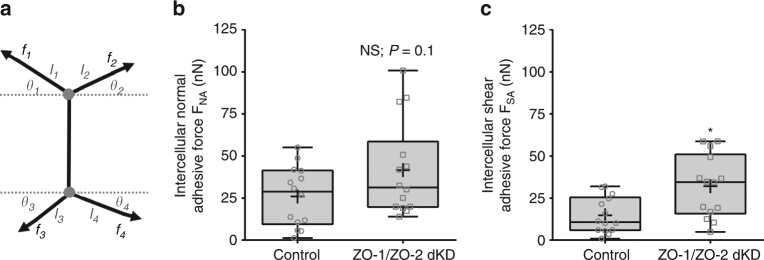
Intercellular adhesion forces level in ZO-1/ZO-2 dKD polarized monolayers are regulated and maintained close to controls. **a** Model of the arrangement of intercellular actomyosin contractile pulling forces and intercellular adhesion forces at a cell–cell contact. The actomyosin pulling forces at tricellular juctions *f*_*i*_ depends on the level of epithelial tension and the length of the cell edge. The intercellular adhesion forces maintain epithelium cohesiveness and depend on actomyosin the level of the pulling forces (*f*_*i*_), cell edge length (*l*_*i*_), and cell edge angle (*θ*_*i*_). **b** Intercellular normal and **c** shear adhesive forces determined on control and ZO-1/ZO-2 dKD polarized epithelial. * indicates significant difference in comparison with control *P* < 0.05(unpaired two-tailed Student’s *t*-test with Welch’s correction); *NS* nonsignificant differences in comparison with control *P* > 0.05(unpaired two-tailed Student’s *t*-test with Welch’s correction); number of measurements pooled for each condition, *n* = 14

Next, to determine the intercellular normal and shear adhesion forces on a confluent epithelium, we consider a sheet of confluent cells comprised of regular hexagons and use Eq. (3) to describe the intercellular pulling force at tricellular junctions. By performing a force balance on a cell–cell junction with two connected tricellular junctions for the net adhesive forces acting in the horizontal and the vertical directions (Fig. 7a), we get:

4 $${F_{{\rm{NA}}}} = \sqrt 3 T\left\{ {{l_2}{\rm{cos}}\left( {{\theta _2}} \right) + {l_4}{\rm{cos}}\left( {{\theta _4}} \right) - {l_1}{\rm{cos}}\left( {{\theta _1}} \right) - {l_3}{\rm{cos}}\left( {{\theta _3}} \right)} \right\},$$

5 $${F_{{\rm{SA}}}} = \sqrt 3 T\left\{ {{l_1}{\rm{sin}}\left( {{\theta _1}} \right) + {l_2}{\rm{sin}}\left( {{\theta _2}} \right) - {l_3}{\rm{sin}}\left( {{\theta _3}} \right) - {l_4}{\rm{sin}}\left( {{\theta _4}} \right)} \right\},$$

where *F*_NA_ is the intercellular normal adhesive force, *F*_SA_ is the intercellular shear adhesive force, *T* is the epithelial tension, *l*_*i*_ is the corresponding cell–cell junction length, and *θ*_*i*_ is the angle of the corresponding cell edge. The main idea of this minimal theoretical model is that intercellular adhesive forces can be determined based on the cortex in-line tensile forces and geometry of polarized epithelial cells.

We next sought to use these equations to measure the intercellular adhesion forces acting within MDCK II polarized monolayers. For determination of intercellular adhesive forces, we needed to measure the cell edges lengths and angles of a pair of adjacent epithelial cells with hexagonal shape within the monolayer (Fig. 7a). To perform these measurements, we fixed MDCK II monolayers after culture for 7–10 days, labeled for E-cadherin, and acquired images using confocal fluorescence microscopy. We extracted the intensity vs. distance profiles for lines that were drawn parallel to individual cell–cell junctions and determined the lengths as previously described. For edges angle measurements, we drew two intersecting lines one parallel to the cell–cell contact and another parallel to the cell edge of interest and measure the angle between the two intersecting lines. Then, we subtracted 90° from the measured angle. These measurements were done to 10 cell pairs depicting a hexagonal shape for both controls and ZO-1/ZO-2 dKD polarized monolayers.

The intercellular adhesive forces of more contractile ZO-1/ZO-2 dKD monolayers is greater compared to controls levels, but this increase seems to be dampened to possibly prevent tissue damage. Calculations of the intercellular adhesive forces show that in ZO-1/ZO-2 dKD the normal adhesive forces were not significantly different whereas the shear adhesive forces were different (Fig. 7b, c). Interestingly, the level of increase in the intercellular adhesive forces is not as large as the increase in pulling forces acting on the tricellular junctions. This is surprising because of the drastic increase in epithelial tension. An explanation for this reduction in the level of force transmission is the dramatic changes in cellular shape and orientation that the more contractile ZO-1/ZO-2 dKD cells undergo (Fig. 6a). Another explanation could be intracellular protein reorganization to reinforce the cell–cell junctions and perijunctional actomyosin belt. A recent study indicated that when paired epithelial cells are being pulled apart, there is a dramatic increase in cytoplasmic E-cadherin, whereas cell–cell adhesion E-cadherin decreased as a function of extension^30^. Although in ZO-1/ZO-2 dKD polarized monolayers there was no change in overall cell E-cadherin protein levels measured by immunoblotting (Supplementary Fig. 7). Taken together, increased intercellular pulling forces at tricellular junctions induce cell shape and orientation changes and intracellular protein reorganization, leading to mechanical reinforcement and reduction in intercellular adhesive forces. A potential explanation may be that E-cadherin not only served as a passive linker between cell–cell junctions and the actomyosin belt, but additionally as an active mechanotransducer to reduce the intercellular forces and maintain epithelial integrity.

## Discussion

In the current study, we demonstrated that several supracellular mechanical properties - epithelial tension, effective viscosity, intercellular forces pulling on the tricellular junctions, and intercellular adhesion forces - can be extracted on polarized monolayers by a noninvasive AFM method compatible with any commercially available AFM system. Our method can measure the supracellular polarized epithelia mechanics limited to the very apical plane (maximum depth ~ 2.5 µm), critical to investigate the mechanics of the adherens junction complex. The method uses FM-AFM frequency shifts recorded at various distances away from the epithelium apical surface to determine epithelial tension. We demonstrated that ZO-1 and ZO-2 depletion in MDCK II cells resulted in elevated epithelial tension consistent with previous studies^11, 12^. The increased epithelial tension is a direct result of reorganization of the actomyosin belt with increased F-actin bundles and myosin II organized in a sarcomeric like distribution. Furthermore, we tested whether myosin II ATPase activity was the major contributor to elevated epithelial tension. Consistent with the work of Fanning et al.^11^ we observed that myosin II contractile ATPase activity, but not ROCK signaling or MLCK activity alone, is the driving force behind the elevated epithelial tension. This is a significant result since it is direct evidence of the dependence of the epithelial tension on actomyosin activity. Altogether, the results show that this simple method is capable of measuring variations in epithelial tension, and that the depletion of ZO-1 and ZO-2 results in elevated apical epithelial tension with myosin II motors ATPase contractile activity being the driving force.

Our results show that the epithelial fluidity changes with alterations to the tight and adherent junctions in polarized epithelium. We demonstrated that alterations of TJ by ZO-1 and ZO-2 depletion in confluent monolayers resulted in elevated epithelial effective viscosity. Additionally, that alteration to the actomyosin dynamics of the perijunctional actin cytoskeleton by inhibition of either myosin II ATPase motor activity, or myosin activation by ROCK or MLCK signaling activity significantly increases the fluidization of the polarized epithelium. Recently, it was shown that confluent monolayers of MDCK II cells adopt more fluid-like mechanical properties after changes in actomyosin dynamics due to inhibition of F-actin polymerization by application of Cytochalasin D^29^. Eventually the cells within the monolayer loosen cell–cell contacts and the epithelial integrity is lost^29^. An explanation for this phenomenon is that because of this fluidization the monolayer is incapable of normal force transmission, an essential process for the maintenance of tissue integrity.

Although intercellular cell–cell force transmission is commonly attributed to the adherens junctions, our results suggest tight junctions also provide an important physical connection with the actomyosin cytoskeleton and contribute to effective epithelial force regulation and transmission. It is known that the actomyosin cytoskeleton exerts tensile forces on E-cadherin^30^. Recently, it was shown that concentrations of P- and E-cadherin were good predictors of intercellular force transmission, with an increased concentration of these two proteins with depletion of ZO-1^31^. The results clearly suggest an increase in intercellular forces with alterations of tight junction structure, consistent with our findings. Although it is possible that a fraction of the ZO proteins are redistributed and play an unappreciated direct role in controlling actin at the AJs. Therefore, an important question emerges. Are the measured forces at levels that will not compromise the integrity of the monolayer? The measured average force required to separate two cells within a MDCK II confluent monolayer was previously shown to be ~1.7 µN^32^, which is about 40 to 120-fold larger than the intercellular adhesive forces measured here. This shows the resilient nature of monolayers to avoid forces that are about 40 to 1200 times larger than the mature intercellular adhesive forces estimated in this work. The intercellular adhesive forces measured in this work are considerably below this limit; however, the ~ 15–42 nN intercellular adhesive force range measured is sufficient to produce openings/pores at the tricellular junctions^11, 12^. Taken together, these observations show a complex picture of cooperation between adherens and tight junctions to regulate monolayer mechanics where cells adhere strongly and pull one another through actomyosin contractility.

While our results focus on the role of ZO proteins and perijunctional actomyosin cytoskeleton to modify the epithelial apical surface mechanics, mid-apical adhesions may also contribute to mechanical changes. As noted previously, our method measures epithelial mechanical properties with depths up to ~ 2.5 µm, meaning that we could potentially measure mid-apical mechanics. By transmission electron microscopy analysis, the tight and adherens junction complexes in MDCK cells are found within 2 µm below the apical surface, most often associated with a desmosome^33^. The lateral cell–cell contacts desmosomes along with AJ provide strong adhesion between cells. They are linked intracellularly to intermediate filaments forming the mid-apical cytoskeleton^34^. It is not known if the intermediate filament cytoskeleton and the actomyosin belt at AJs are linked and if so how. However, because of the strong adhesion, contribution to force transmission and epithelial mechanical strength has not been ruled out. Therefore, future studies using the proposed method can be used to determine to what extend whether any desmosomes may contribute to changes in epithelial apical surface mechanics.

In our future studies, a potential application for the method described here is to measure epithelial tension, fluidity, and intercellular adhesive forces in the inner ear organ of Corti sensory epithelium to study the auditory mechanics contributions to hearing loss. The organ of Corti is one of the key organs responsible in converting vibrations into sound. It is known that tensional homeostasis in the organ of Corti is critical for hearing integrity. It has been shown that mutations on nonmuscle myosin II leads to hearing loss in humans^35^. For example, Ebrahim et al.^36^ showed that bipolar nonmuscle myosin II minifilaments form a continuous contractile sarcomeric like belt around each epithelial cell in the organ of Corti. Inhibition of myosin II activity relaxes the sensory epithelium and presumably affects the force transmission along the junctional perimeter of each cell impacting the tensional homeostasis of the epithelial tissue^36^. Recently, it has been shown that novel mutations in the tight junction protein tricellulin are linked to hearing loss in humans^37^. When tricellulin is truncated in the mouse, in the organ of Corti sensory epithelium cells shows reduced binding of tricellulin to ZO-1^37^. This reduction in binding between tricellulin to ZO-1 may significantly impact the force transmission capacity of the sensory epithelium. Therefore, we strongly believe our method can be used to provide additional critical insight to the importance of epithelial tension to study tissue morphogenesis and hearing loss.

A potential point for improvement of this method that may introduce artifacts to the magnitude of the measured apical surface epithelial mechanics is the fact that we used a piezoelectric material to mechanically vibrate the base of the micron size cantilever. This is the most popular excitation method because virtually all commercially available AFM systems provide this capability. It is well known that the piezo excitation tuning spectrum in liquids is filled with many apparent spurious resonance peaks that are not directly related to the cantilever natural resonance^38^. This makes it difficult to determine the natural frequency of the cantilever. A reasonable solution to this issue is to choose the largest resonance peak closest to the natural frequency of the cantilever predetermined by thermally driven spectrum, improving the acquisition of the true cantilever dynamics, as we did here (see Methods and Supplementary Fig. 8). Supplementary Figure 8b piezo-actuated excitation spectra (amplitude and phase response curves) show that the method does not introduce significant artifacts since the cantilever resonance peak at smaller gaps is visibly well-defined with no significant change in shape or location of peaks, thus ruling out the existence of spurious peaks. Nonetheless, we recommend for future work combining this method with a direct cantilever excitation such as magnetic, Lorentz force, or photothermal excitation to more sensitively record the resonance frequency of the cantilever.

In summary, our method can measure the supracellular tension, effective viscosity, and intercellular adhesive forces at the very apical plane (~ 2.5 µm) in polarized epithelia. Our data indicate that ZO proteins play a role in regulating the apical mechanical properties of polarized epithelium. Specifically, depletion of ZO-1 and ZO-2 in MDCK II polarized monolayers elevates the apical surface epithelial tension, effective viscosity, and the pulling forces at tricellular junctions, but the intercellular adhesion forces shows a more complex scenario. These results reveal a potential new mechanism in which polarized monolayers can relieve or reduce excessive load that could potentially damage the monolayer integrity.

## Methods

### Cell lines

Madin Darby Canine Kidney (MDCK) II Tet-off cells (clone T23; Clontech, Mountain View, CA) were cultured under standard conditions at 37 °C and 5% CO_2_ in high-glucose Dulbecco’s Modified Eagle’s Medium (DMEM (4.5 g l^−1^ glucose); Life Technologies, Carlsbad, CA) supplemented with 10% Tet-tested Fetal Bovine Serum (FBS; Atlanta Biologicals, Flowery Branch, GA), Penicillin-Streptomycin (Life Technologies). Cells were plated at a density of 10^5^ cells cm^−2^ on glass-bottom petri dishes (Willco Wells, Amsterdam, The Netherlands) and cultured for 7–10 days to obtain 100% confluence, with media changes every 2–3 days.

To make ZO-1/ZO-2 double knockdown cell lines (ZO-1/ZO-2 dKD, clone 3B3; Fanning et al.^11^), we first generated ZO-1 single knockdown cells by cotransfection of pSVZeo (Invitrogen, Carlsbad, CA) with a mixture of three different ZO-1 shRNAs cloned into the pSuper vector (Oligoengine, Seattle, WA); stable cell lines were selected in 1 mg ml^−1^ zeocin (InvivoGen, San Diego, CA). Antibiotic-resistant clonal cell lines were screened by immunoblotting and immunofluorescence^10^. The ZO-1 single knockdown cells were cotransfected with plasmid pBlast49 and pSUPER ZO-2 shRNAs and stable lines were selected in standard media supplemented with 10 μg ml^−1^ blasticidin (InvivoGen, San Diego, CA). Antibiotic-resistant clones were screened for both ZO-1 and ZO-2 depletion by immunoblot and immunofluorescence. To generate a Tet-inducible full-length ZO-1 rescue construct (ZO1R), the previously described pTRE-ZO1myc transgene^39^ was modified by QuikChange Multi Site-Directed mutagenesis (Agilent Technologies, Santa Clara, CA) to disrupt the shRNA-binding sites. It was necessary to make conservative mutations at two distinct sites in the myc-tagged ZO-1 transgene to ensure that all possible interactions with the three different shRNA sequences were disrupted. The resulting pTRE ZO1R construct was cotransfected with the plasmid pTK-hygro into the ZO-1/ZO-2 double knockdown cells as described above. Clones were selected in 200 μg ml^−1^ hygromycin B (Sigma Aldrich, St. Louis, MO) and inducible rescue determined by immunoblot and immunofluorescent analysis. ZO-1 rescue construct expression was repressed by addition of 50 ng ml^−1^ doxycycline to media and induced by doxycycline removal.

### Pharmacological manipulation

To test the role of myosin II in the epithelial tension of polarized epithelium, we treated monolayers of MDCK II Tet-off cells and ZO-1/ZO-2 dKD cells for 15–20 h with 100 µM of the myosin II activity inhibitor blebbistatin (Toronto Research Chemicals, North York, Ontario, Canada), 30 µM of the ROCK inhibitor Y-27632 (Sigma Aldrich), or 2 µM of the MLCK activity inhibitor ML-7 (Sigma Aldrich). To block the expression of full-length ZO-1 rescue on ZO1R cells we treated monolayers with 50 ng ml^−1^ doxycycline (Sigma Aldrich) and incubate for 2–3 days. To induce full-length ZO-1 rescue on ZO1R cells we washed out multiple times monolayers previously treated with doxycycline and incubate for additional 1–2 days.

### Atomic force microscopy

Cells were cultured for at least 7 days on a glass-bottom dish (Willco Wells) before experiments. For atomic force microscopy, samples measurements were performed using a Bruker Bioscope Catalyst AFM system (Bruker, Santa Barbara, CA) mounted on an inverted Axiovert 200 M microscope system (Carl Zeiss, Gottingen, Germany) equipped with a Confocal Laser Scanning Microscope 510 Meta (LSM 510 Meta, Carl Zeiss) and a 40x (0.6 NA, Plan-Apochromat) objective lens (Carl Zeiss). The AFM biological system was placed on a vibration isolation table (Kinetic Systems, Boston, MA). A heating stage (Bruker) was used to maintain physiological temperature 37 °C of cells during measurements. Modified AFM microcantilevers with an attached microsphere were obtained from Novascan (Novascan, Ames, IA). The triangular silicon nitride cantilevers had a 20, 25, or 35 µm polystyrene bead attached to the free end. The optical deflection sensitivity of each cantilever was obtained by performing a force-distance curve on a stiff glass surface. The spring constant were obtained by using thermal tune method^40^ built in the AFM system. Calibrated spring constants for cantilevers were 0.7–1.5 N m^−1^. Note, except for Supplementary Figs. 3 and 4, all other experiments presented in this study were performed using cantilevers with attached 25 µm bead.

Once the sample was placed in the AFM X-Y stage, the cantilever was positioned in liquid far from the sample surface and allowed to thermally equilibrate. For noncontact frequency modulation AFM (FM-AFM)^21^ experiments, tapping mode AFM was engaged. Immediately, the cantilever tune mode was launched to choose the driving frequency. An initial frequency sweep was performed to locate *f*_*π*/2_. Because for our experiments we are using piezo driven excitation in liquids a forest of peak is observed^38^. We chose the largest peak found in the vicinity of the cantilever natural frequency typically 32–38 kHz, Supplementary Fig. 8. Next, the cantilever was approached and gently placed in contact with the cells monolayer apical surface. Then, the cantilever tune mode was launched and initially set to position the microsphere of the cantilever 6 µm above the monolayer (a crucial feature of the cantilever tune mode is that enables the positioning of the cantilever sphere at a controlled height over the sample surface with nanometer resolution) and the phase lag between the piezo and the cantilever was set *π*/2. Additionally, the drive oscillatory amplitude of the piezo was adjusted to ensure the cantilever oscillation amplitude at *f*_*π*/2_ was below 10 nm. Frequency sweeps were recorded with a 10 kHz frequency range around *f*_*π*/2_. Next, frequency sweeps were recorded for multiple distances between the bead and the monolayer apical surface from 6 µm down to 500 nm using 500 nm intervals. Lastly, the vibrating cantilever was moved 10 µm away for the surface and a final frequency sweep was recorded to provide *f∞*. Immediately after each FM-AFM set of measurements, the ramp mode was engaged and the tip was brought in contact with the apical surface. Five force-distance curves were recorded using 3 µm ramp distance with ~ 10 nN applied force and ~ 1 µm indentation at 1 Hz.

### Analysis of atomic force microscopy data

All computations were performed using MATLAB software (The Mathworks, Natick, MA). For FM-AFM, the phase-frequency response curves from each recorded frequency sweep were extracted. Then, each the phase-frequency curves obtained at different distances from the sample surface were fitted with a second-order polynomial on the vicinity of *π*/2 to determine *f*_*π*/2_. The phase-frequency curves slope d*Φ/*d*f* was computed using the frequency sweeps recorded at 2 µm above the sample by fitting the phase-frequency curves with a third-order polynomial. Estimates of d*Φ/*d*f* did not change between the measured height range over the epithelium (Supplementary Fig. 9). *∆f*_*π*/2_-height curves were reconstructed and fitted with the epithelial tension equation, Eq. (1) using nonlinear least squares method to best fit the data.

For quasi-static force-distance AFM, the elastic Young’s modulus (*E*; Pa) was computed by fitting each force-distance curve with the Hertz contact mechanics model for indenting an infinite isotropic elastic half-space with a solid sphere^41^:

6 $${F_{{\rm{Hertz}}}} = \frac{4}{3}\frac{E}{{\left( {1 - {v^2}} \right)}}\sqrt R {\delta ^{3/2}},$$

where *F* is the applied force, *υ* is the Poisson’s ratio assumed to be 0.5, *R* is the sphere radius, and *δ* is the sample mean indentation.

### Confocal immunofluorescence microscopy

Tet-off MDCK II (wild-type and ZO-1/ZO-2 dKD cells) were cultured on glass-bottom dishes for 7–10 days, rinsed in ice cold Dulbecco’s Phosphate Buffered Saline (dPBS; Life Technologies) and fixed in 1% paraformaldehyde. Then cells were washed twice in dPBS, permeabilized with 0.2% Triton X-100 (EMD Biosciences, San Diego, CA) for 10 mins, quenched with 50 mM NH_4_Cl and blocked in 2% normal goat serum (Life Technologies) in dPBS for 60 min. Cells were incubated with antibodies for E-cadherin (24E10; Cell Signaling Technologies, Beverly, MA) at 1:100 dilution and ZO-1 rat monoclonal antibody 40.76 (homemade^42^) at 1:50 dilution for 60 min. Dishes were washed four times in block solution and incubated with 1:2000 dilution of anti-Rat Cy2 (712-225-150) and anti-Rabbit Cy3 (15-165-166) species-specific affinity-purified conjugated secondary antibodies (both from Jackson Immunoresearch Laboratories, West Grove, PA) and Alexa Fluor 647-phalloidin (A22287; Life Technologies) with 1:40 dilution. Filters were washed 4 times, rinsed in H_2_O and mounted with mowiol (Calbiochem) supplemented with 1% *N*-propylgallate.

Samples were imaged on a Zeiss LSM 780 confocal microscope (Carl Zeiss) using a 63x (1.4 NA, Plan-Apochromat) objective lens (Carl Zeiss). Photomultiplier settings were identical for all conditions (controls and dKD cells) to allow direct comparison. Z-stacks images were acquired through the whole cell volume with a fixed pinhole of 0.7 µm (all channels) and a step size of 0.35 µm. All confocal Z-stacks images were edited using the open access image analysis software Fiji (http://fiji.sc/Fiji)^43^. Confocal Z-stack images are presented as maximum intensity Z-projections of 1.05 µm (three frames) final depth. Final images were assembled in Adobe Illustrator 10 (Adobe Systems Incorporated, San Jose, CA) software.

### Immunoblot assay

Cells were plated at 5 × 10^4^ onto 12 mm transwell filter inserts (Corning, Cambridge, MA) and grown for 7 days in high glucose DMEM, 10% FBS, and penicillin/streptomycin (all from Life Technologies). In some cases, cells were treated (uninduced) or not (induced) with 50 ng ml^−1^ doxycycline (Sigma Aldrich). Filters were washed twice in ice-cold Dulbecco’s phosphate-buffered saline (dPBS; Life Technologies) and lysed in 0.150 ml of 2× SDS loading buffer (4% SDS, 125 mM Tris pH 6.8, 20% glycerol, 0.3 M β-mercaptoethanol and 0.002% bromophenol blue). Samples were sonicated briefly to disrupt genomic DNA, heated to 95 °C for 3 min, and resolved by SDS–PAGE. Gels were transferred to nitrocellulose filters and then blocked in a solution of PBS (Life Technologies) and 10% non-fat dry milk powder (NFDM) for 1 h. Filters were incubated for 1 h in primary antibodies rat ZO-1 monoclonal (1:500; homemade^42^), Ms E-cadherin (24E10; 1:1000; Cell Signaling Technologies), rabbit non-muscle myosin 2B (909901; 1:1,000; BioLegend, San Diego, CA), and rabbit ZO-2 (38-9100; 1:1000; ThermoFisher Scientific) diluted in a solution of PBS, 5% NFDM, and 1% Tween-20 (PBS-T); washed four times for 5 min each in PBS-T; and incubated for 30 min with 1:2,000 dilution of the appropriate species-specific secondary antibodies anti-rat 680 (612-144-120), anti-mouse 680 (610-730-124), and anti-rabbit 800CW (611-731-127) (Rockland, Gilbertsville, MD). After four more washes in PBS-T, filters were imaged with the Odyssey infrared imaging system (Licor Biosciences, Lincoln, NE).

### Cell–cell junction length and angle measurements

Confocal images of fixed and stained MDCK II monolayers were analyzed to determine cell edges length and angles using the image analysis software Fiji. For cell edge measurement, a straight line was drawn on the edge of interest to measure the length. We capture the intensity vs. distance profiles were captured for lines that were drawn parallel to individual cell–cell junctions using Fiji. The forward difference of the intensity profiles, which approximates of the derivative, was calculated for each line. The lengths of the intensity curves for E-cadherin fluorescence were then determined by measuring the distance between the vertices on the intensity vs. distance profiles. This provides an unbiased measure spanning the cell–cell junction. To measure the cell edge angle, the built-in angle tool in Fiji software was used. For angle measurements, we drawn two intersecting lines one parallel to the cell–cell contact and another parallel to the cell edge of interest and measure the angle between the two intersecting lines. Lastly, we subtract 90° to the angle measured using the angle tool to extract the cell edges angle.

### Statistical analysis

One-way ANOVA and unpaired two-tailed student’s *t* with Welch’s correction tests were performed using the software GraphPad Prism 6 (GraphPad Software, San Diego, CA).

### Code availability

A computer code implemented in MATLAB was used for the analysis of acquired FM-AFM data to extract the epithelial tension and effective viscosity. The computer code is available from the corresponding author upon reasonable request.

### Data availability

The data supporting the findings of this study are available from the corresponding author upon reasonable request.

## Electronic supplementary material

Supplementary Information

## References

[1] Harris TJC, Tepass U (2010). Adherens junctions: from molecules to morphogenesis. Nat. Rev. Mol. Cell Biol..

[2] Shen L, Weber CR, Raleigh DR, Yu D, Turner JR (2011). Tight junction pore and leak pathways: a dynamic duo. Annu. Rev. Physiol..

[3] Getsios S, Huen AC, Green KJ (2004). Working out the strength and flexibility of desmosomes. Nat. Rev. Mol. Cell Biol..

[4] Kumar NM, Gilula NB (1996). The gap junction communication channel. Cell.

[5] Guillot C, Lecuit T (2013). Mechanics of epithelial tissue homeostasis and morphogenesis. Science.

[6] Capaldo CT, Macara IG (2007). Depletion of E-cadherin disrupts establishment but not maintenance of cell junctions in madin-darby canine kidney epithelial cells. Mol. Biol. Cell.

[7] Leerberg JM (2014). Tension-sensitive actin assembly supports contractility at the epithelial zonula adherens. Curr. Biol..

[8] Spracklen AJ, Peifer M (2015). Actin and apical constriction: some (Re)-assembly required. Dev. Cell.

[9] Anderson, J. M. & Van Itallie, C. M. Physiology and function of the tight junction. *Cold Spring Harbor Perspectives in Biology* **1**, a002584 (2009). 10.1101/cshperspect.a002584 PMC2742087 20066090

[10] Van Itallie CM, Fanning AS, Bridges A, Anderson JM (2009). ZO-1 stabilizes the tight junction solute barrier through coupling to the perijunctional cytoskeleton. Mol. Biol. Cell.

[11] Fanning AS, Van Itallie CM, Anderson JM (2012). Zonula occludens-1 and -2 regulate apical cell structure and the zonula adherens cytoskeleton in polarized epithelia. Mol. Biol. Cell.

[12] Choi W (2016). Remodeling the zonula adherens in response to tension and the role of afadin in this response. J. Cell Biol..

[13] Heisenberg C-P, Bellaïche Y (2013). Forces in tissue morphogenesis and patterning. Cell.

[14] Logue, J. S. et al. Erk regulation of actin capping and bundling by Eps8 promotes cortex tension and leader bleb-based migration. *eLife* **4**, e08314 (2015). 10.7554/eLife.08314 PMC4522647 26163656

[15] Michael M (2016). Coronin 1B reorganizes the architecture of F-actin networks for contractility at steady-state and apoptotic adherens junctions. Dev. Cell.

[16] Cartagena-Rivera AX, Wang W-H, Geahlen RL, Raman A (2015). Fast, multi-frequency, and quantitative nanomechanical mapping of live cells using the atomic force microscope. Sci. Rep.

[17] Cartagena-Rivera AX, Logue JS, Waterman CM, Chadwick RS (2016). Actomyosin cortical mechanical properties in nonadherent cells determined by atomic force microscopy. Biophys. J..

[18] Krieg M (2008). Tensile forces govern germ-layer organization in zebrafish. Nat. Cell. Biol..

[19] Pietuch A, Brückner BR, Janshoff A (2013). Membrane tension homeostasis of epithelial cells through surface area regulation in response to osmotic stress. Biochim. Biophys. Acta.

[20] Rouven Brückner B, Pietuch A, Nehls S, Rother J, Janshoff A (2015). Ezrin is a major regulator of membrane tension in epithelial cells. Sci. Rep..

[21] Gavara N, Chadwick RS (2010). Noncontact microrheology at acoustic frequencies using frequency-modulated atomic force microscopy. Nat Methods.

[22] Chadwick RS, Liao Z (2008). High-frequency oscillations of a sphere in a viscous fluid near a rigid plane. SIAM Rev..

[23] Chadwick RS, Cartagena-Rivera AX (2015). Using noncontact AFM frequency shifts to determine stereocilia bundle stiffness and tension in the developing cochlear sensory epithelium. AIP Conf. Proc..

[24] Iyer S, Gaikwad RM, Subba Rao V, Woodworth CD, Sokolov I (2009). Atomic force microscopy detects differences in the surface brush of normal and cancerous cells. Nat Nano.

[25] Kovács M, Tóth J, Hetényi C, Málnási-Csizmadia A, Sellers JR (2004). Mechanism of blebbistatin inhibition of myosin II. J. Biol. Chem..

[26] Hirose M (1998). Molecular dissection of the Rho-associated protein kinase (p160ROCK)-regulated neurite remodeling in neuroblastoma N1E-115 cells. J. Cell Biol..

[27] Watanabe T, Hosoya H, Yonemura S (2007). Regulation of myosin II dynamics by phosphorylation and dephosphorylation of its light chain in epithelial cells. Mol. Biol. Cell.

[28] Blanchard GB (2009). Tissue tectonics: morphogenetic strain rates, cell shape change and intercalation. Nat Methods.

[29] Brückner BR, Nöding H, Janshoff A (2017). Viscoelastic properties of confluent MDCK II cells obtained from force cycle experiments. Biophys. J..

[30] Borghi N (2012). E-cadherin is under constitutive actomyosin-generated tension that is increased at cell–cell contacts upon externally applied stretch. Proc. Natl Acad. Sci. USA.

[31] Bazellières E (2015). Control of cell–cell forces and collective cell dynamics by the intercellular adhesome. Nat. Cell Biol..

[32] Harris AR (2012). Characterizing the mechanics of cultured cell monolayers. Proc. Natl Acad. Sci. USA.

[33] Stevenson BR, Anderson JM, Goodenough DA, Mooseker MS (1988). Tight junction structure and ZO-1 content are identical in two strains of Madin-Darby canine kidney cells which differ in transepithelial resistance. J. Cell Biol..

[34] Garrod D, Chidgey M (2008). Desmosome structure, composition and function. Biochim. Biophys. Acta.

[35] Lalwani AK (2000). Human nonsyndromic hereditary deafness DFNA17 Is due to a mutation in nonmuscle myosin MYH9. Am. J. Hum. Genet..

[36] Ebrahim S (2013). NMII forms a contractile transcellular sarcomeric network to regulate apical cell junctions and tissue geometry. Curr. Biol..

[37] Riazuddin S (2006). Tricellulin is a tight-junction protein necessary for hearing. Am. J. Hum. Genet..

[38] Putman CAJ, Van der Werf KO, De Grooth BG, Van Hulst NF, Greve J (1994). Tapping mode atomic force microscopy in liquid. Appl. Phys. Lett..

[39] Fanning AS (2007). The unique-5 and -6 Motifs of ZO-1 regulate tight junction strand localization and scaffolding properties. Mol. Biol. Cell.

[40] Butt HJ, Jaschke M (1995). Calculation of thermal noise in atomic force microscopy. Nanotechnology..

[41] Hertz H (1882). Ueber die Berührung fester elastischer Körper. J. Für Die Reine Angew. Math..

[42] Anderson JM, Stevenson BR, Jesaitis LA, Goodenough DA, Mooseker MS (1988). Characterization of ZO-1, a protein component of the tight junction from mouse liver and Madin-Darby canine kidney cells. J. Cell. Biol..

[43] Schindelin J (2012). Fiji: an open-source platform for biological-image analysis. Nat Methods.

