# Supplementary material for: Apical surface supracellular mechanical properties in polarized epithelium using noninvasive acoustic force spectroscopy
Source: Nat Commun. 2017 Oct 18;8:1030. doi: 10.1038/s41467-017-01145-8 (PMC5715111; doi:10.1038/s41467-017-01145-8)
Supplement: Supplementary file 1 — Supplementary Information [file 41467_2017_1145_MOESM1_ESM.pdf]

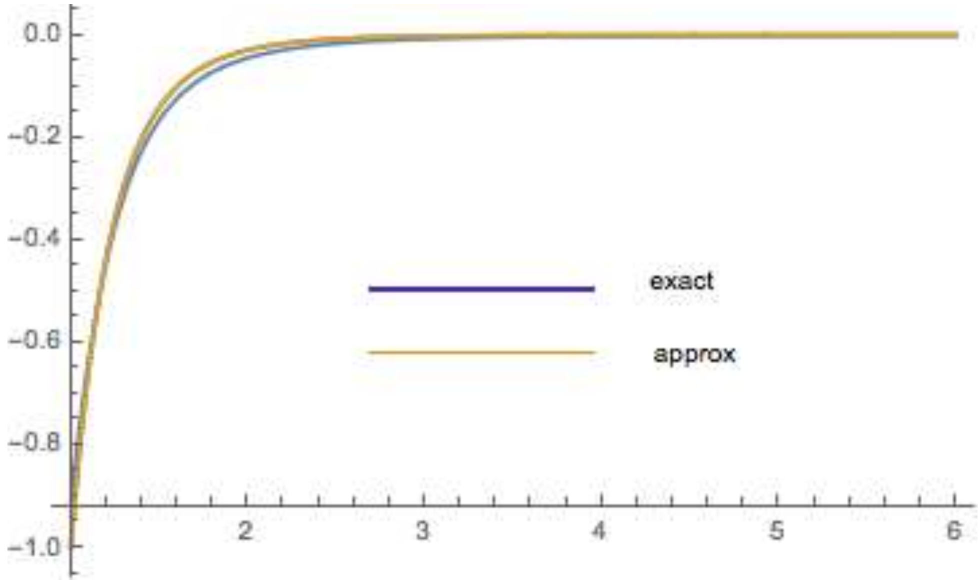

Supplementary Figure 1. The simplified model provides accurate solutions. Plotted is the imaginary part of the pressure vs. the scaled radial coordinate. As you can see the comparison between the approximate and "exact" solution is very good. The pressure at the origin overestimated by 6% and the force is underestimated by 9.5%.

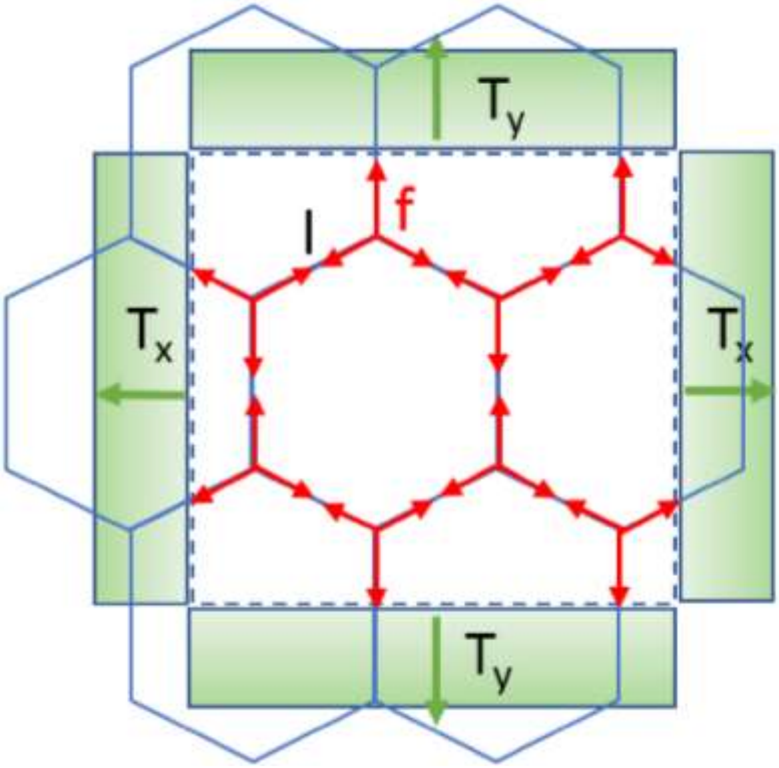

Supplementary Figure 2. Model for apical surface epithelial tension development by myosin II contraction at hexagonal edges in polarized epithelium. Consider a sheet of confluent epithelial cells comprised of regular hexagons with cell edge l. We assume that myosin molecular motors exerts an intercellular pulling force f away from each tricellular junction because of the fact that myosin exerts a contractile force along the hexagon sheets lateral walls (actomyosin belt). The myosin contraction creates an isotropic tension field $T_x = T_y$.

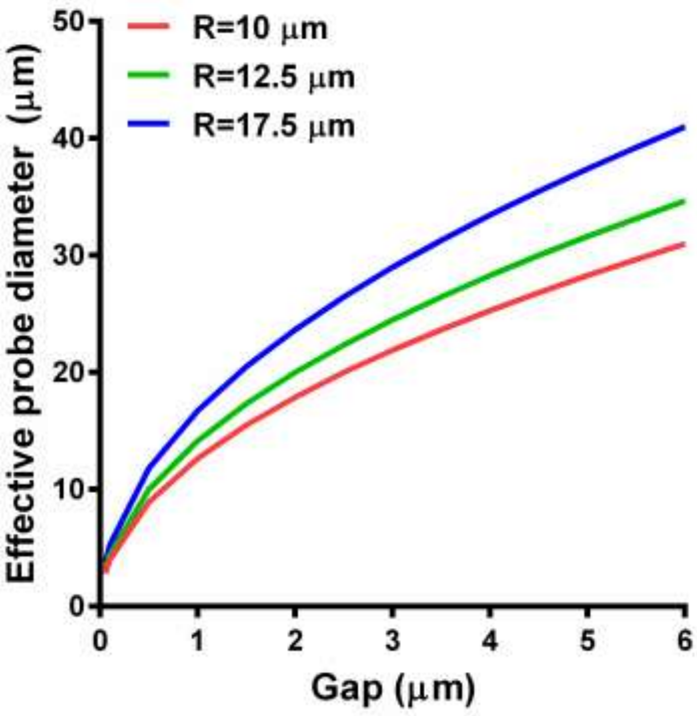

Supplementary Figure 3. Calculation of effective probe diameter by different bead sizes at multiple distances from the tissue surface. Effective probe diameter calculated $(D_{eff} = 4(\mathrm{R}h_m)^{1/2})$ for beads with size (R= 10 µm, red line; R=12.5 µm, green line; R=17.5 µm, blue line) showing that for the distances we positioned the vibrating bead from the tissue we probe the large-scale mechanics. Typical single cell apical surface cell diameter measured by ImageJ on immunofluorescence images in a confluent MDCK II monolayer is ~ 10 µm.

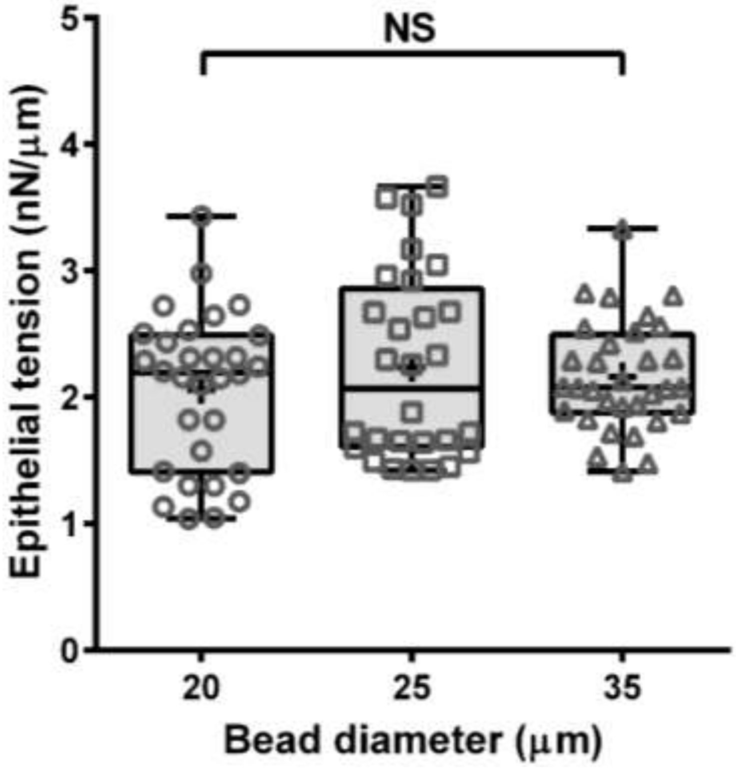

Supplementary Figure 4. Determination of apical surface epithelia tension in MDCK II polarized monolayers using different bead sizes There are not significant differences on the epithelial tension in MDCK II monolayers for the beads tested. In summary; 20 µm T=2.1 ± 0.6 nN µm^{-1}, 25 µm T=2.2 ± 0.7 nN µm^{-1}, and 35 µm T=2.2 ± 0.4 nN µm^{-1}. Data are represented as mean ± standard deviation; *NS* indicates nonsignificant differences in comparison with 25 µm bead size control P>0.05(unpaired two-tailed student's *t*-test with Welch's correction).; number of measurements pooled for 2-3 independent experiments for each condition, n= 30, 28, and 32 for cantilevers with attached bead diameter 20 µm, 25 µm, and 35 µm, respectively.

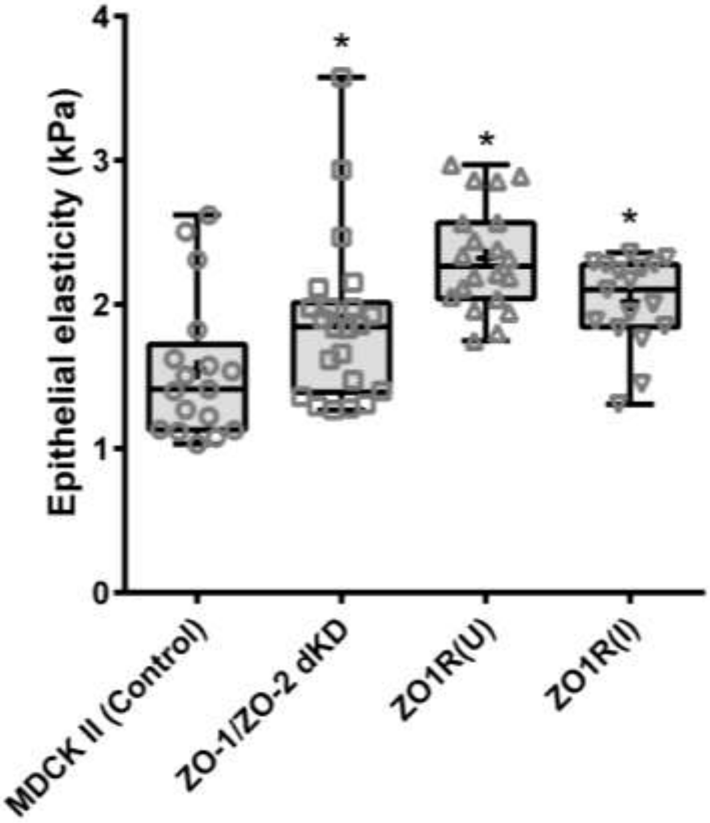

Supplementary Figure 5. Elastic Young's modulus measured on MDCK II Tet-off cell lines using standard quasi-static force-distance curves. Hertz contact mechanics model for spherical indenters was used. In ZO-1/ZO-2 dKD in MDCK II Tet-off cells the apical surface elastic Young's modulus dramatically increases. In ZO1R(U) dKD cells the apical elastic modulus is significantly elevated when compared to control, but the elastic modulus is partially restored when ZO1R(I) dKD cells are expressing a Tet-inducible full-length ZO-1 rescue transgene. U, (+dox, uninduced); I, (-dox, induced). In summary; MDCK II (control) E=1.547 ± 0.5 kPa, ZO-1/ZO-2 dKD E=1.872 ± 0.56 kPa, ZO1R(U) E=2.322 ± 0.37 kPa, and ZO1R(I) E=2.019 ± 0.31 kPa. Data are represented as mean ± standard deviation; * indicates significant different in comparison with control P<0.05 (unpaired two-tailed student's t-test with Welch's correction); number of measurements pooled for 2-3 independent experiments for each condition, n= 20, 22, 25, and 22 for control, ZO-1/ZO-2 dKD, ZO1R(U), and ZO1R(I), respectively.

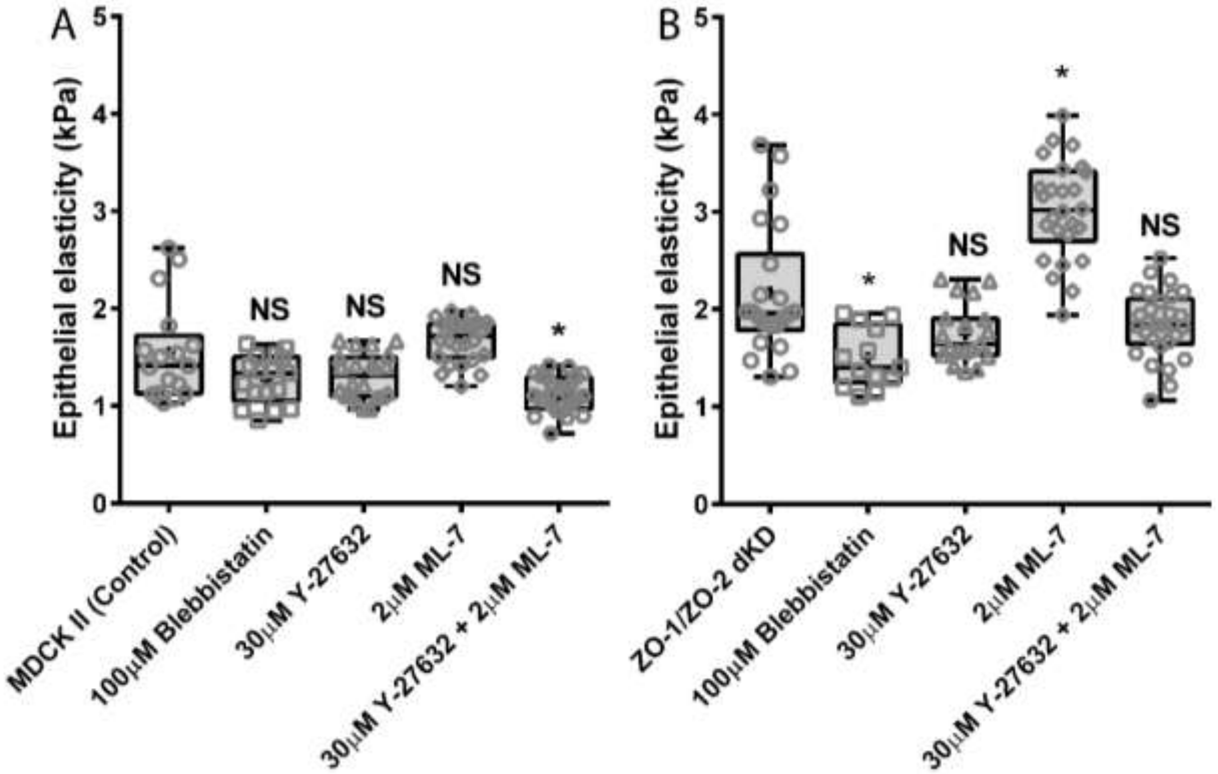

Supplementary Figure 6. Elastic Young's modulus measured on MDCK II Tet-off cell lines threated with 100 µM blebbistatin, 30 µM Y-27632, and 2 µM ML-7. Hertz contact mechanics model for spherical indenters was used. Confluent monolayers of MDCK II controls and ZO-1/ZO-2 dKD cells were treated with 100 µM blebbistatin, 30 µM Y-27632, 2 µM ML-7, or 30 µM Y-27632 + 2 µM ML-7 pharmacological drugs for 15 to 20 hours. **(A)** The plot shows the epithelial tension for MDCK II control untreated polarized monolayers, and treated either with 100 µM blebbistatin, 30 µM Y-27632, 2 µM ML-7, or 30 µM Y-27632 + 2 µM ML-7. **(B)** The plot shows the epithelial tension for MDCK II ZO-1/ZO-2 dKD untreated polarized monolayers, and treated either with 100 µM blebbistatin, 30 µM Y-27632, 2 µM ML-7, or 30 µM Y-27632 + 2 µM ML-7. Myosin II activity inhibition dramatically reduces the epithelium tension, whereas inhibition of MLCK or ROCK signaling do not cause a significant change on epithelial tension. In summary; MDCK II (control) E=1.55 ± 0.5 kPa, 100 µM blebbistatin E=1.27 ± 0.25 kPa, 30 µM Y-27632 E=1.3 ± 0.24 kPa, 2 µM ML-7 E=1.68 ± 0.22 kPa, 30 µM Y-27632 + 2 µM ML-7 E=1.12 ± 0.18 kPa, ZO-1/ZO-2 dKD E=2.17 ± 0.68 kPa, 100 µM blebbistatin E=1.5 ± 0.31 kPa, 30 µM Y-27632 E=1.752 ± 0.31 kPa, 2 µM ML-7 E=3 ± 0.5 kPa, 30 µM Y-27632 + 2 µM ML-7 E=1.84 ± 0.34 kPa,. In **(A-B)** data are represented as mean ± standard deviation; * indicates significant different in comparison with control P<0.05 (unpaired two-tailed student's t-test with Welch's correction); *NS* indicates nonsignificant differences in comparison with control P>0.05 (unpaired two-tailed student's t-test with Welch's correction); number of measurements pooled for 2-3 independent experiments for each condition, n= 20, 27, 22, 26, and 27 for control, 100 µM blebbistatin, 30 µM Y-27632, 2 µM ML-7, and 30 µM Y-27632 + 2 µM ML-7, respectively, and n= 22, 15, 19, 26, and 29 for ZO-1/ZO-2 dKD, 100 µM blebbistatin, 30 µM Y-27632, 2 µM ML-7, and 30 µM Y-27632 + 2 µM ML-7, respectively.

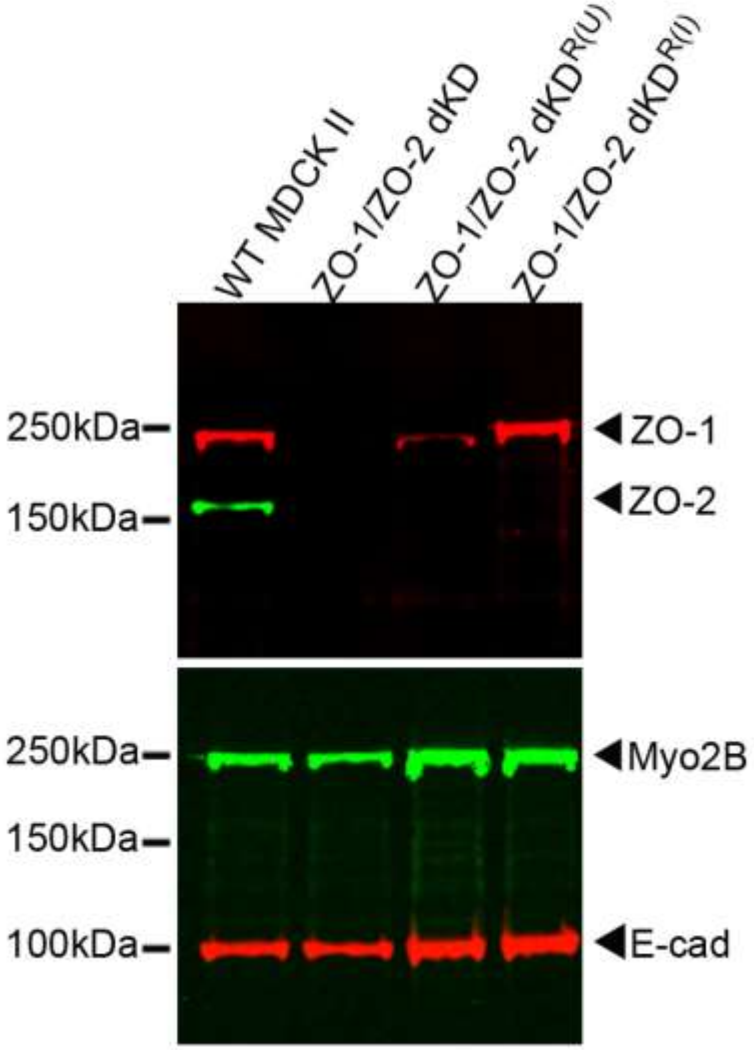

Supplementary Figure 7. Immunoblot shows ZO-1 and ZO-2 proteins are effectively depleted in dKD cell lines, whereas E-cadherin and myosin II protein levels are equal to controls. Western blot of ZO-1 and ZO-2 polypeptides in MDCK II Tet-off control cells, ZO-1/ZO-2 dKD cells, and ZO1R dKD cells expressing a Tet-inducible full-length ZO-1 rescue transgene. ZO-1 and ZO-2 expression in dKD cell lines are almost undetectable. In ZO1R dKD cell line, induction of ZO1R (I) restores ZO-1 expression to levels equivalent to those in controls, while ZO-2 expression remains suppressed. Myosin IIB and E-cadherin expression in dKD cell lines are equal to control cells.

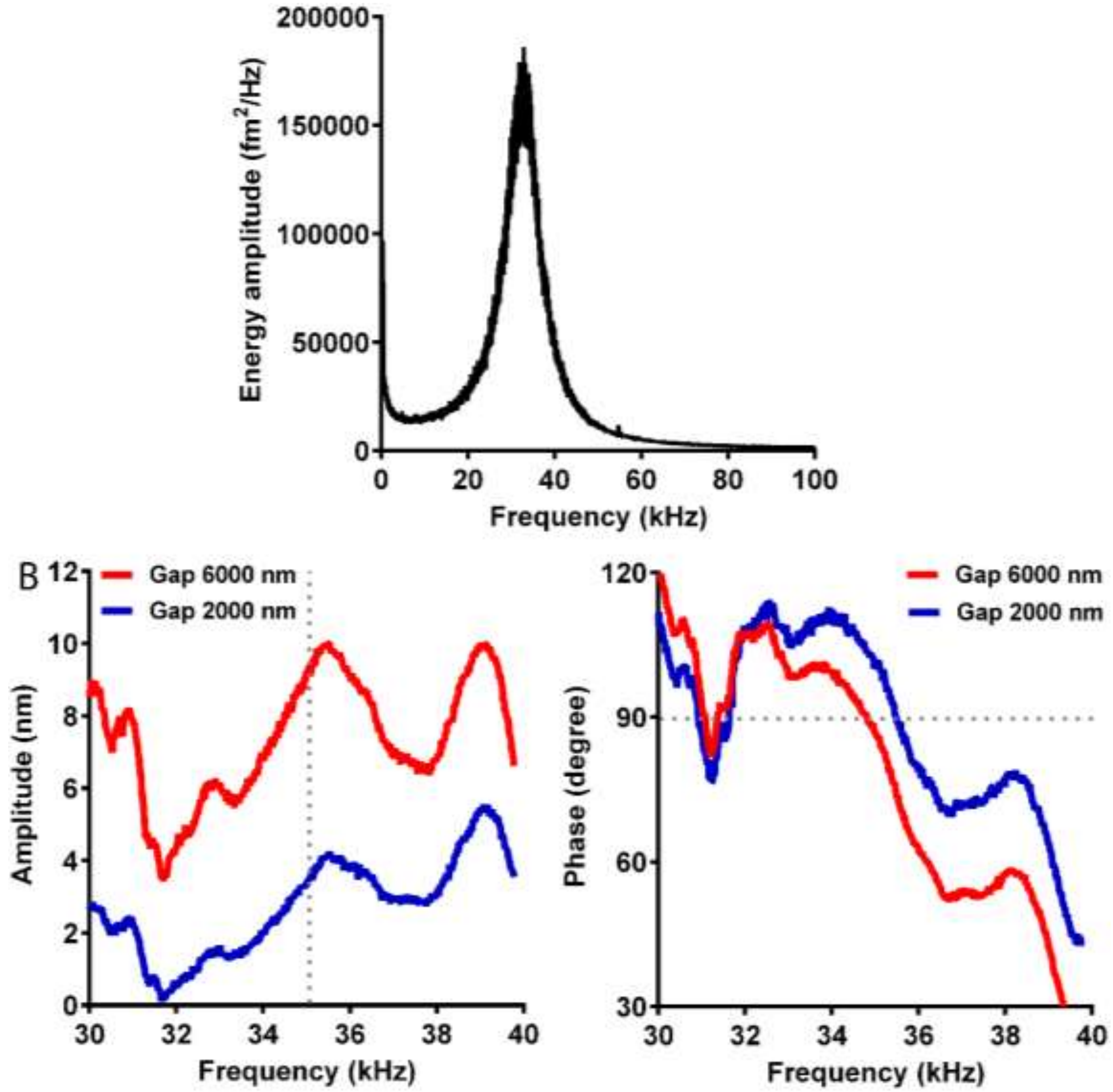

Supplementary Figure 8. Determination of driving frequency for noninvasive acoustic FM-AFM measurements. (A) Thermal tune of cantilever with microsphere in liquids depicting the cantilever first flexural eigenmode resonance frequency. (B) Same cantilever typical tune curve for piezo-driven excitation in liquids showing "forest of peaks". Amplitude of oscillation and phase lag curves recorded at 6 μm and 2 μm over the polarized monolayer. To choose the driving frequency, we compared both curves and select the largest peak in the tune curve forest of peak that is the closest to the resonance frequency measured by thermal tune. Horizontal dotted line in phase response curve corresponds to π/2 phase lag between the piezo and the cantilever, this $f_{\pi/2}$ is set as the driving frequency. Vertical dotted line in amplitude response curve corresponds to the selected driving frequency. The amplitude response curves show that the method does not introduce significant artifacts since the cantilever resonance peak at smaller gaps is visibly well-defined with no significant change in or location of peaks, thus ruling out the existence of spurious peaks.

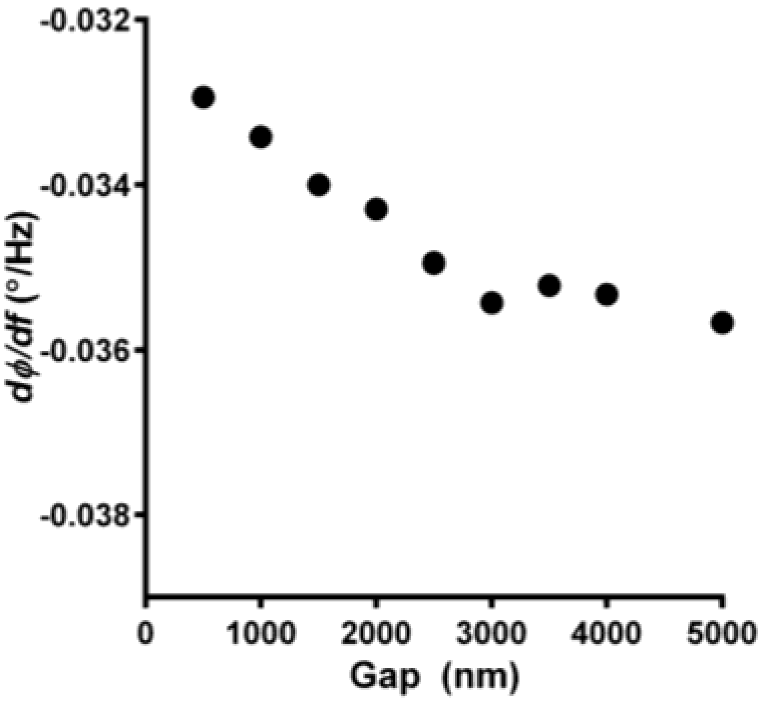

Supplementary Figure 9. Changes in the slope of the phase against frequency response curve. When the acoustically oscillating micron size sphere is moved closer to the MDCK II WT polarized epithelium apical surface changes in $d\phi/df$ measured at $f_{\pi/2}$ are negligible.

Supplementary Note 1

<u>Theory for frequency shift and epithelial tension relationship for compliant substrates</u>

For tipless cantilevers in fluids oscillating near rigid substrates it has been previously shown that dissipative loading is important for resonant frequency changes, whereas inertial loading is weak[1]. In contrast, on compliant substrates inertial and dissipative loadings are important[2]. Consider a rigid micron-sized sphere of radius R (m) attached to the end of a microcantilever with calibrated spring constant k_c (N m^{-1}) that undergoes small oscillations with acoustic frequency ω (rad s^{-1}) in an incompressible fluid bath with density ρ (kg m^{-3}) and viscosity μ (Pa-s). The oscillating sphere approaches a flat or curved compliant substrate. It is assumed that the sphere oscillation amplitude is very small compared to the minimum gap height h_m (m), and that h_m, in turn is small compared to the sphere radius R. The oscillatory motion is normal to the nearby compliant substrate characterized by the substrate stiffness parameter K (N m^{-3}) and radius of curvature R_s.

Calculation of gap pressure

Lubrication theory for linearized unsteady Stokes equations for fluid motion applies for conditions where the sphere oscillation amplitude is much smaller than the minimum gap height h_m and the sphere radius R. The motion is axisymmetric, thus we can describe the induced flow in terms of the Stokes stream function $\Psi(r, z)$, where r is the radial distance measured from the vertical axis of revolution through the minimum gap, and z denotes the distance along the normal axis measured upward from the midpoint of the two surfaces. The critical idea behind lubrication of thin gaps is that radial derivatives are small compared to the axial derivatives[3]. This allows us to neglect the radial derivatives compared to axial in equation for Stokes stream function $\Psi(r, z)$. The radial velocity u and the axial velocity w are expressed in terms of $\Psi(r, z)$:

$$\boldsymbol{u = \frac{1}{r}\frac{\partial \Psi}{\partial z}; w = -\frac{1}{r}\frac{\partial \Psi}{\partial r}} \text{. (Eq. 1)}$$

The resulting equation is:

$$\boldsymbol{\left(\frac{d^2}{dz^2} - \gamma^2\right)\frac{d^2\Psi}{dz^2} = 0; -\gamma^2 = i\frac{\rho\omega}{\mu} = i\frac{1}{\delta^2}} \text{. (Eq. 2)}$$

where γ is a parameter related to the inverse of the unsteady boundary layer thickness δ. Using parabolic approximation for the geometry of the model and no-slip boundary conditions for viscous fluids, the boundary conditions are $u=w=0$ on the compliant substrate located at $z = z_l = -(1/2)h_m - (1/2)(r^2/R)+\ldots$, and $u=0$ and $w=-V_0$ located on the approaching rigid sphere at $z = z_u = (1/2)h_m + (1/2)(r^2/R_s)+\ldots$. In terms of the stream function these conditions are equivalent to:

$$\boldsymbol{on\ z = z_l: \frac{\partial \Psi}{\partial z} = 0; \Psi = 0} \text{ , (Eq. 3)}$$

$$\boldsymbol{on\ z = z_u}: \frac{\partial \Psi}{\partial z} = \mathbf{0}; \boldsymbol{\Psi} = \frac{1}{2} \boldsymbol{r^2 V_0}$$, (Eq. 4)

on the compliant substrate and rigid sphere, respectively. Eq. 3 approximates the substrate as rigid. Later this approximation is relaxed. The theory that proceeds is a self-consistent perturbation theory, since the deformation of the substrate ~10 nm is small compared to the gap.

A general solution for the stream function has the form:

$$\boldsymbol{\Psi} = \mathbf{A} + \mathbf{B}(\boldsymbol{z - z_l}) + \boldsymbol{C} \cosh \boldsymbol{\gamma}(\boldsymbol{z - z_l}) + \boldsymbol{D} \sinh \boldsymbol{\gamma}(\boldsymbol{z - z_u})$$. (Eq. 5)

The four boundary conditions result in a 4X4 linear system to solve for the 4 constants (***A, B, C,* and *D***).

The radial velocity profile in the gap turns out to be:

$$\boldsymbol{U(r, z)} = \frac{1}{2} \boldsymbol{V_0 r \gamma} \left\{ \frac{-\cosh \Phi + \frac{\cosh \Phi - 1}{\sinh \Phi} \sinh \gamma(z - z_l) + \cosh \gamma(z - z_u)}{\cosh \Phi \left(2 \frac{\cosh \Phi - 1}{\sinh \Phi} - \Phi\right)} \right\}$$, (Eq. 6)

where $\Phi = \gamma(z_u - z_l) = \gamma h_m(1 + (r^2/(2h_m R^*)) + \cdots)$. Let $1/R^* = 1/R + 1/R_s$, where $R^*$is defined as the effective radius when two spherical surfaces are interacting. Note that this generalized approach was initially developed for compliant curved substrates; however, since our research work presented in this paper is on flat polarized monolayers we need to define R^* for flat substrates. Thus, $R^* = R$ (bead radius) if the substrate is flat.

We note the radial flux is given by:

$$\int_{z_l}^{z_u} \boldsymbol{U(r, z) dz} = \frac{1}{2} \boldsymbol{V_0 r}$$. (Eq. 7)

In addition, we need to differentiate the radial velocity twice with respect to *z*, yielding the relation:

$$\left.\frac{\partial^2 U(r,z)}{\partial z^2}\right|_{z_l} = \frac{\frac{1}{4} V_0 r \gamma^3}{\left(\tanh\left(\frac{1}{2}\Phi\right) - \frac{1}{2}\Phi\right)}$$. (Eq. 8)

Equations Eq. 7 and Eq. 8 have assumed that the substrate is rigid so far. To consider a deformable substrate we introduce a new correction function *a(r)* to multiply V_0 and we will use a radial momentum and continuity of mass to eliminate this function in terms of *K*, which is stiffness per unit area normal to the deformable substrate. The linearized radial momentum equation relates the radial gradient of pressure *P* (N m^{-2}) to viscous shear along the substrate:

$$\left.\frac{1}{\mu} \frac{\partial P}{\partial r}\right|_{z = z_l} = \left.\frac{\partial^2 U(r,z)}{\partial z^2}\right|_{z_l} = \frac{\frac{1}{4} V_0 r \gamma^3 a(r)}{\left(\tanh\left(\frac{1}{2}\Phi\right) - \frac{1}{2}\Phi\right)}$$. (Eq. 9)

We now balance the radial outflow and the radial inflow due to the moving rigid sphere and the axial outflow due to the movement of the deformable substrate using the continuity of mass equation:

$$\boldsymbol{Q_{out} = 2\pi r \int_{z_l}^{z_u} U(r,z)dz = \pi r^2\, a(r) V_0}\text{, (Eq. 10)}$$

$$\boldsymbol{Q_{in} = \pi r^2 V_0}\text{ , (Eq. 11)}$$

$$\boldsymbol{Q_s = 2\pi \int_0^r v(\rho)\rho d\rho;\ v(r) = -i\omega \frac{p(r)}{K}}\text{ . (Eq. 12)}$$

The mass conservation can be written as $Q_{in} = Q_{out} - Q_s$:

$$\boldsymbol{\pi r^2 V_0 = \pi r^2 a(r) V_0 - \frac{2\pi i\omega}{K}\int_0^r p(\rho)\rho d\rho}\text{ . (Eq. 13)}$$

Now differentiate with respect to *r* and use Eq. 9:

$$\boldsymbol{2rV_0 = \frac{d}{dr}\left(\frac{4}{\mu}\frac{\left(\tanh\left(\frac{1}{2}\Phi\right)-\frac{1}{2}\Phi\right)}{\gamma^3} r \frac{dp}{dr}\right) - \frac{2i\omega r}{K} p(r)}\text{ . (Eq. 14)}$$

Finally, the resulting pressure equation is:

$$\boldsymbol{\frac{1}{r}\frac{d}{dr}\left(r\left(\tanh\left(\frac{1}{2}\Phi\right) - \frac{1}{2}\Phi\right)\frac{dp}{dr}\right) + \frac{i\omega}{2K}\mu\gamma^3 p(r) = \frac{\mu\gamma^3}{2} V_0}\text{ . (Eq. 15)}$$

The parameter Φ is proportional to the ratio of the gap h_m to the boundary layer thickness δ, which turns out to be small for the conditions of the experiment ~ 1/40. For $\Phi \ll 1$ then:

$$\boldsymbol{\left(\tanh\left(\frac{1}{2}\Phi\right) - \frac{1}{2}\Phi\right) \cong -\frac{1}{24}\ \Phi^3 + O(\Phi)^5}\text{ . (Eq. 16)}$$

Keeping the first term, the simplified pressure equation is:

$$\boldsymbol{\frac{1}{r}\frac{d}{dr}\left(r\left(1 + \frac{r^2}{2Rh_m}\right)^3 \frac{dp}{dr}\right) - \frac{12i\mu\omega}{Kh_m^3} p(r) = -\frac{12\mu}{h_m^3} V_0}\text{ . (Eq. 17)}$$

Scale *r* by letting $r^* = \frac{r}{\sqrt{2Rh_m}}$:

$$\boldsymbol{\frac{1}{r^*}\frac{d}{dr}\left(r^*\left(1 + r^{*2}\right)^3 \frac{dp}{dr^*}\right) - \frac{24i\mu R\omega}{Kh_m^2} p(r) = -\frac{24\mu R}{h_m^2} V_0}\text{ . (Eq. 18)}$$

Additionally, scale *p* by letting $p^* = \frac{p\omega}{V_0 K}$ to get the resulting dimensionless pressure equation:

$$\boldsymbol{\frac{1}{\kappa r^*}\frac{d}{dr^*}\left(r^*\left(1 + r^{*2}\right)^3 \frac{dp^*}{dr^*}\right) - ip^* = -1}\text{ . (Eq. 19)}$$

where κ denotes the dimensionless parameter $\kappa = \frac{24\omega\mu R}{K h_m^2}$ that turns out to be the ratio of tangential to normal stress acting on the substrate. It is important to note that κ is different than K.

To further simplify the pressure equation in search of a closed form solution, we let $H^* = 1 + r^{*2}$ and $\frac{d}{dr^*} = \frac{dH^*}{dr^*}\frac{d}{dH^*}$. Introducing this transformation into Eq. 19 gives:

$$\boldsymbol{\frac{4}{\kappa}\frac{d}{dH^*}\left(H^{*3}(H^* - 1)\frac{dp^*}{dH^*}\right) - ip^* = -1} \text{ . (Eq. 20)}$$

We can get an approximate closed form solution by assuming $H^* \gg 1$ (not valid at the origin). Therefore, we solve:

$$\boldsymbol{\frac{4}{\kappa}\frac{d}{dH^*}\left(H^{*4}\frac{dp^*}{dH^*}\right) - ip^* = -1} \text{ . (Eq. 21)}$$

The rationale behind this assumption is that we would like a closed form solution to fit experimental data, thus we are willing to give up some accuracy in order to obtain a simple analytical closed form expression.

We can eliminate the dimensionless parameter κ with further scaling by letting $H^* = \frac{1}{2}\sqrt{i\kappa}h$ and solve the homogenous equation:

$$\boldsymbol{\frac{d}{dh}\left(h^4\frac{dp^*}{dh}\right) - p^* = 0} \text{ . (Eq. 22)}$$

Using Mathematica software, we find the decaying solution:

$$\boldsymbol{p^* = C_1\left\{\sinh\left(\frac{1}{h}\right) - \frac{1}{h}\cosh\left(\frac{1}{h}\right)\right\}} \text{ . (Eq. 23)}$$

Evaluating C_1 by requiring $p^* = -i$ and $H^* = 1$ at $h = \frac{2}{\sqrt{i\kappa}}$ yields:

$$\boldsymbol{C_1 = \frac{-i}{\sinh\left(\frac{1}{2}\sqrt{i\kappa}\right) - \frac{1}{2}\sqrt{i\kappa}\cosh\left(\frac{1}{2}\sqrt{i\kappa}\right)}} \text{ . (Eq. 24)}$$

This choice of boundary condition at the origin is motivated by noting $p^* = -i$ is a particular solution. To determine the accuracy of Eq. 23 we matched the solution of Eq. 23 to the Bessel function $I_0\left[\left(i\kappa(H^* - 1)\right)^{1/2}\right]$, which is accurate near the origin. Plotted in Supplementary Fig. 1 is the imaginary part of the pressure vs the scaled radial coordinate. The comparison between the approximate and "exact" solution is very good. The pressure at the origin overestimated by 6% and the force is underestimated by 9.5%.

Calculation of force on sphere

We calculate the force F on the sphere by calculating the force on the substrate, since the latter can be obtained in closed form. We can estimate the error by noting the pressure gradient across the gap is small. The axial momentum is a balance between the pressure gradient, viscous stress, and fluid inertia. The ratio of the third to the second term is $\omega h^2/\upsilon$ ~ 0.2 in our experiment, so actually the axial pressure gradient is more affected by normal viscous stress than fluid inertia. Therefore, we can estimate $\Delta p/p \sim R\mu\omega^2/(8k_c\Delta f)$ ~ 0.126, which is an acceptable error. Proceeding with the calculation of force:

$$\boldsymbol{F = 2\pi \int_0^\infty prdr = \frac{2\pi V_0 K}{\omega} \int_0^\infty p^* rdr} \text{ . (Eq. 25)}$$

Substituting $rdr = 2Rh_m r^* dr^* = Rh_m dH^* = \frac{1}{2}\sqrt{i\kappa}Rh_m dh$ into Eq. 25 yield:

$$\boldsymbol{F = \frac{\pi V_0 K}{\omega} \sqrt{i\kappa} R h_m \int_{2/\sqrt{i\kappa}}^\infty p^* dh} \text{ . (Eq. 26)}$$

Using Mathematica software to evaluate the integral yields:

$$\boldsymbol{F = \frac{\pi V_0 K}{\omega} \sqrt{i\kappa} R h_m C_1 \left\{1 - \frac{2}{\sqrt{i\kappa}} \sinh\left(\frac{1}{2}\sqrt{i\kappa}\right)\right\}} \text{ . (Eq. 27)}$$

So, the change in force ΔF as the cantilever with the micron-sphere moves toward the surface from a far location h_{far} is:

$$\boldsymbol{\Delta F = \frac{\pi V_0 K}{\omega} \sqrt{i\kappa} R \left(h_m - h_{far}\right) C_1 \left\{1 - \frac{2}{\sqrt{i\kappa}} \sinh\left(\frac{1}{2}\sqrt{i\kappa}\right)\right\}} \text{ . (Eq. 28)}$$

We now consider two approximations involving κ that can be used to simplify the equation and solve for the frequency shift.

- if $\kappa \ll 1$ (small κ approximation):

Note that; $\sinh\epsilon = \epsilon + \frac{1}{6}\epsilon^3 + \cdots$ and $\cosh\epsilon = 1 + \frac{1}{2}\epsilon^2 + \cdots$, then $1 - \frac{2}{\sqrt{i\kappa}}\sinh\left(\frac{1}{2}\sqrt{i\kappa}\right) \sim \frac{i\kappa}{24}$. Additionally, expanding the hyperbolic terms of Eq. 21 we get that:

$$\boldsymbol{C_1 = \frac{-i}{-\frac{1}{16}\sqrt{i\kappa}(i\kappa)} = \frac{16}{\kappa\sqrt{i\kappa}}} \text{ . (Eq. 29)}$$

Substituting Eq. 29 into Eq. 28 results in:

$$\boldsymbol{\Delta F = \frac{\pi V_0 K}{\omega} \sqrt{i\kappa} R \left(h_m - h_{far}\right) \frac{16}{\kappa\sqrt{i\kappa}} \frac{i\kappa}{24} = i\omega V_0 \left\{\frac{2}{3} \frac{\pi K R\left(h_m - h_{far}\right)}{\omega^2}\right\}} \text{. (Eq. 30)}$$

- if $\kappa \gg 1$ (large κ approximation applicable to the present work):

Note that for the case $\kappa \gg 1$; $sinh \to cosh$; and $C_1 \to \frac{-i}{e^{\frac{\sqrt{i\kappa}}{2}}\left(1-\frac{\sqrt{i\kappa}}{2}\right)} \to \frac{i}{\frac{\sqrt{i\kappa}}{2}e^{\frac{\sqrt{i\kappa}}{2}}}$. Then substituting these relations into Eq. 27 yield:

$$\Delta \boldsymbol{F} = \frac{\boldsymbol{\pi V_0 K}}{\boldsymbol{\omega}}\sqrt{\boldsymbol{i\kappa}}\boldsymbol{R}\left(\boldsymbol{h_m} - \boldsymbol{h_{far}}\right)\left(\frac{\boldsymbol{i}}{\frac{\sqrt{\boldsymbol{i\kappa}}}{2}}\right)\left(\frac{\boldsymbol{-1}}{\frac{\sqrt{\boldsymbol{i\kappa}}}{2}}\right) = -\frac{\boldsymbol{i\pi V_0 K}}{\boldsymbol{\omega}}\boldsymbol{R}\left(\boldsymbol{h_m} - \boldsymbol{h_{far}}\right)\frac{\boldsymbol{4}}{\sqrt{\boldsymbol{i\kappa}}} =$$

$$-\boldsymbol{i\omega V_0}\left\{\frac{\boldsymbol{2\sqrt{2}KR\left(h_m - h_{far}\right)\pi(1-i)}}{\boldsymbol{\sqrt{\kappa}\omega^2}}\right\} \text{ . (Eq. 31)}$$

Calculation of frequency shift

Following Chadwick & Liao[3] who treated the sphere, cantilever, and fluid as a simple oscillator:

$$\boldsymbol{k_c = M\omega^2} \text{ . (Eq. 32)}$$

where *M* is the sum of the mass of the sphere-cantilever and the hydrodynamic added mass, and *ω* is the near frequency peak where the sphere motion lags the piezo motion by a phase of π/2. The relation holds independently of the damping of the system. When the gap between the sphere and the compliant sample changes by moving the cantilever, only the hydrodynamic mass and the resonant frequency change, while the cantilever spring constant remains unchanged (assuming that tip-sample interaction forces do not come into play). Therefore,

$$\boldsymbol{\Delta k_c = 0 = \Delta M\omega^2 + 2M\omega\Delta\omega} \text{ . (Eq. 33)}$$

And using Eq. 32 to eliminate *M*, it follows that:

$$\frac{\boldsymbol{\Delta\omega}}{\boldsymbol{\omega}} = \frac{\boldsymbol{\Delta f}}{\boldsymbol{f}} = \frac{\boldsymbol{-\Delta M\omega^2}}{\boldsymbol{2k_c}} \text{ . (Eq. 34)}$$

where $\Delta\omega$ is the frequency shift resulting from a change in the hydrodynamic mass ΔM, due to a wall squeeze film effect that depends on the compliance of the wall. The change in hydrodynamic mass ΔM can be obtained from the force change Eq. 28; ΔM is the component of force proportional to the acceleration of the oscillating sphere. Thus, the relationships describing frequency shift for both *κ* cases are:

- if $\kappa \ll 1$ (small *κ* approximation):

From the total force equation Eq. 30 we can obtain the change in hydrodynamic mass as $\Delta M = \frac{2}{3}\frac{\pi KR(h_m - h_{far})}{\omega^2}$. Substituting ΔM into Eq. 34 yield:

$$\boldsymbol{\Delta f} = \frac{\boldsymbol{\pi KRf(h_m - h_{far})}}{\boldsymbol{3k_c}} \text{ . (Eq. 35)}$$

- if $\kappa \gg 1$ (large *κ* approximation):

From the force change equation Eq. 31 we can obtain the change in hydrodynamic mass as $\Delta M = \frac{2\sqrt{2}KR(h_{far}-h_m)\pi}{\sqrt{\kappa}\omega^2}$. Substituting ΔM into Eq. 34 yield:

$$\Delta f = \frac{\sqrt{\pi f R}(h_{far}-h_m)h_m K^{3/2}}{2k_c\sqrt{6\mu}} \text{. (Eq. 36)}$$

Tension and substrate stiffness parameter (T-K) relations for compliant membranes

We will follow the development given in the work of Chadwick & Cartagena-Rivera[4], but using the new formulas obtained herein. Here we assume that the compliant substrate can be modeled by a sheet having an isotropic pretension T (N m^{-1}). Now the substrate stiffness parameter K (N m^{-3}) must be related to the sheet tension T using membrane deformation theory. First, we use polar coordinates *(r, θ)* and consider the vertical displacement w of a horizontal membrane due to a vertical point force F at the origin:

$$w(r,0) = \frac{F}{2\pi T} log\left(\frac{r}{r_0}\right) \text{. (Eq. 37)}$$

This is the Green's function[5] for a deformable membrane under tension and it gives the singular radially symmetric solution to Laplace's equation, which governs the vertical deformation of the membrane. The membrane displacement vanishes at $r=r_0$ that we choose to be the effective probe radius, where the pressure effectively vanishes. Integrate Eq. 37 over the membrane to obtain the displacement due to a radially symmetric pressure distribution *p(r)*:

$$\frac{p_o}{K} = w(0,0) = \frac{1}{2T}\int_0^{r_0} p(r) log\left(\frac{r^2}{r_0^2}\right) r dr \text{. (Eq. 38)}$$

where p_0 is the pressure at the origin. To evaluate Eq. 38 we take r_0 be the effective probe radius, so $r_0^2 = 4Rh_m$ and we then introduce the transformations $r^{*2} = \frac{r^2}{r_0^2}$, $u = r^{*2}$, $du = \frac{2}{r_0^2} r dr$, and $F(u) = \frac{p(u)}{p_0}$. Now introducing these expressions into Eq. 38 yield:

$$\frac{T}{K} = \frac{Rh_m}{p_0}\frac{V_0 K}{\omega}\int_0^1 F(u) log(u)\, du. \text{ (Eq. 39)}$$

where $F(u) = sinh\frac{\sqrt{i\kappa}}{2(1+u)} - \frac{\sqrt{i\kappa}}{2(1+u)} cosh\frac{\sqrt{i\kappa}}{2(1+u)}$.

There are two approximations that can be used to simplify the equation Eq. 39 to solve for the tension and substrate stiffness parameter relationship,

- if $\kappa \ll 1$ (small κ approximation):

$$F(u) \sim -\frac{1}{3}\left(\frac{\sqrt{i\kappa}}{2(1+u)}\right)^3 \text{. (Eq. 40)}$$

Then substituting Eq. 40 into Eq. 39 yields,

$\boldsymbol{\frac{T}{K} = \frac{Rh_m}{p_0}\frac{V_0 K}{\omega}\left(-\frac{1}{3}\left(\frac{1}{2}\sqrt{i\kappa}\right)^3\right)\int_0^1 \frac{log(u)}{(1+u)^3}du.}$ (Eq. 41)

Using Mathematica software to solve the integral in Eq. 41 $\int_0^1 \frac{log(u)}{(1+u)^3}du = -\frac{1}{4}(1+\log(4))$, recalling that $p_0 = -\frac{iV_0K}{\omega}$, substituting both results into Eq. 41, and solving for the dominant real solution yield the simple analytical *T-K* relation:

$\boldsymbol{\frac{T}{K} = \frac{Rh_m(1+\log(4))}{192\sqrt{2}}\kappa^{3}/_{2},}$ (Eq. 42)

- if *κ≫1* (large *κ* approximation):

$\boldsymbol{F(u) \sim -\frac{\sqrt{i\kappa}}{4(1+u)}e^{\frac{\sqrt{i\kappa}}{2(1+u)}}}$. (Eq. 43)

Because $log(u)$ is singular when evaluated at the origin, the integral is dominated by the value of the integrand at the origin:

$\boldsymbol{F_0 = F(0) = \left(-\frac{1}{4}\sqrt{i\kappa}\right)e^{\frac{1}{2}\sqrt{i\kappa}}}$ **.** (Eq. 44)

Substituting these expressions into Eq. 39:

$\boldsymbol{\frac{4T}{KRh_m} = \sqrt{i\kappa}e^{\frac{1}{2}\sqrt{i\kappa}}}$ **.** (Eq. 45)

Rearranging Eq. 45 yield to the following simple expression $\left|\frac{4T}{KRh_m}\right| = \sqrt{\kappa}e^{--\frac{1}{2\sqrt{2}}\sqrt{\kappa}}$. For the special case when $\kappa \gg 1$, $\sqrt{\kappa}e^{-\frac{1}{2\sqrt{2}}\sqrt{\kappa}} \sim 1$. Therefore, this simplification yields the simple analytical *T-K* relation:

$\boldsymbol{\frac{T}{K} = \frac{Rh_m}{4}}$. (Eq. 46)

Viscoelastic tissue

Gavara & Chadwick previously developed an approach to estimate the viscous dissipation of a compliant substrate using noncontact frequency-modulated atomic force microscopy[2]. In summary, they measured the slope of the phase-frequency curve to determine the ratio of the elastic and viscous forces in the compliant substrate;

$\boldsymbol{\frac{\omega c}{K} = -\frac{6}{f\frac{d\phi}{df}}}$, (Eq. 47)

where c is the substrate three-dimensional damping parameter, ϕ is the cantilever response phase, and $\frac{d\phi}{df}$ the slope of the phase-frequency curve.

Substitute Eq. 47 into Eq. 46;

$$\boldsymbol{c} = -\frac{\mathbf{12}\boldsymbol{T}}{\boldsymbol{R}\boldsymbol{h_m}}\frac{\mathbf{1}}{\boldsymbol{\pi}\boldsymbol{f}^{\mathbf{2}}\frac{\boldsymbol{d\phi}}{\boldsymbol{df}}} = \frac{\mu_{eff}}{\lambda}, \text{ (Eq. 48)}$$

where μ_{eff} is the substrate effective viscosity (Pa-s) and $\lambda = (T/K)^{1/2} = 1/2(Rh_m)^{1/2}$ is the penetration depth. Thus, the effective viscosity can be determined from;

$$\boldsymbol{\mu_{eff}} = -\frac{\mathbf{6}\boldsymbol{T}}{(Rh_m)^{1/2}}\frac{\mathbf{1}}{\boldsymbol{\pi}\boldsymbol{f}^{\mathbf{2}}\frac{\boldsymbol{d\phi}}{\boldsymbol{df}}}\text{ . (Eq. 49)}$$

Analysis of experimental data

An extremely important step is how to use the obtained mathematical relations to effectively calculate the epithelial tension by fitting the experimental data obtained using an atomic force microscope (AFM). The AFM actually measures the phase lag between the piezo and the cantilever when vibrating acoustically and brought to close proximity to the membrane surface. Our theory says that in order to measure the frequency shift, the phase lag need to be kept constant at π/2.

To determine the tension on a confluent monolayer, we substitute Eq. 46 into Eq. 36 to obtain a simple relation between frequency shift and tension:

$$\Delta\boldsymbol{f} = \frac{\mathbf{4}}{\boldsymbol{k_c}\boldsymbol{R}}\sqrt{\frac{\boldsymbol{\pi}\boldsymbol{f}\boldsymbol{h_m}\boldsymbol{T}^{\mathbf{3}}}{\mathbf{6}\boldsymbol{\mu}}}\left(\frac{\boldsymbol{h_{far}}}{\boldsymbol{h_m}} - \mathbf{1}\right)\text{, (Eq. 50)}$$

where $\Delta f = f_{near} - f_{far}$ is the frequency shift (Hz) where the phase is π/2; f_{far} (Hz) is the "unperturbed" cantilever resonance frequency far away from the sample surface where the phase is π/2; f_{near} (Hz) is the "perturbed" cantilever resonance frequency near the sample surface where the phase is π/2; k_c (N m^{-1}) is the cantilever spring constant; R (m) is the radius of the cantilever microsphere; h_{far} (m) is the farthest distance between the lowest portion of the sphere and the epithelial apical surface where the cantilever dynamics are unperturbed; h_m (m) is the minimum distance between the lowest portion of the sphere and the membrane surface; μ (Pa-s) is the incompressible fluid viscosity; and T (N m^{-1}) is the epithelial tension.

To successfully fit the frequency shift-tension relation model to frequency shift-gap curves we generated a MATLAB (The Mathworks, Natick, MA) code. First, the experimental phase response between the piezo and cantilever curves was analyzed to extract the frequency shift at each gap. Then, the frequency shift-gap curves were reconstructed. After curve reconstruction, we used nonlinear least squares method to best fit the data and determine the tension. The code effectively solves the nonlinear least squares data fitting problems by using a predefined starting

point x_0 and finding a minimum of the sum of squares for the function of interest. Because we want a rigorous way to analyze the data we used goodness of fit statistical method to calculate the R^2 value and an estimate of the error variance. We pursue the best R^2 value.

Supplementary Note 2

Calculation of intercellular adhesion forces

We will follow the development described in the work of Chadwick & Cartagena-Rivera[4] to determine the intercellular adhesion forces on a confluent epithelium. Consider a sheet of confluent cells comprised of regular hexagons with cell edge *l*, as shown in Supplementary Fig. 2 we assume that myosin molecular motors exert an intercellular pulling force f away from each tricellular junction since myosin exerts a contractile force along the hexagon sheet lateral walls (actomyosin belt). The myosin contraction creates a tension field, T_x and T_y acting on the dotted rectangle, which is the unit periodic lattice of width $W = (2)3^{3/2}l$ and height $H = 3l$. A force balance in the *y* and *x* directions yields the following equations:

$\boldsymbol{T_y W = 2f}$, (Eq. 51)

$\boldsymbol{T_x H = \sqrt{3}f}$. (Eq. 52)

Equations 51 and Eq. 52 relate the intercellular pulling forces *f* to the tensions T_x and T_y, respectively. Note $T_x/T_y = 1$, i.e. the tension field is isotropic. The myosin contractile force and the epithelial tension *T* are then related by the simple equation:

$\boldsymbol{f = \sqrt{3}Tl}$, (Eq. 53)

This myosin pulling force at the tricellular junction can be used to determine the intercellular adhesion forces. The main text schematic diagram Fig. 7A depicts the pulling forces acting within each tricellular junction on an isolated cell-cell junction within the epithelium. Then, by performing force balance for the forces acting in the horizontal direction, we get:

$\boldsymbol{F_{NA} = f_2 cos(\theta_2) + f_4 cos(\theta_4) - f_1 cos(\theta_1) - f_3 cos(\theta_3)}$. (Eq. 54)

Now substituting Eq. 53 into Eq. 54 yield the equation for the intercellular normal adhesion force:

$\boldsymbol{F_{NA} = \sqrt{3}T\{l_2 cos(\theta_2) + l_4 cos(\theta_4) - l_1 cos(\theta_1) - l_3 cos(\theta_3)\}}$, (Eq. 55)

where F_{NA} is the intercellular normal adhesion force, l_i is the corresponding cell edge length, and θ_i is the angle of the corresponding cell edge.

Similarly, by performing force balance for the forces now acting in the vertical direction, we get:

$\boldsymbol{F_{SA} = f_1 sin(\theta_1) + f_2 sin(\theta_2) - f_3 sin(\theta_3) - f_4 sin(\theta_4)}$. (Eq. 56)

Now substituting Eq. 53 into Eq. 56 yield the equation for the intercellular shear adhesion force F_{SA}:

$$\boldsymbol{F_{SA}} = \sqrt{\mathbf{3}}\boldsymbol{T}\{\boldsymbol{l_1 sin(\theta_1)} + \boldsymbol{l_2 sin(\theta_2)} - \boldsymbol{l_3 sin(\theta_3)} - \boldsymbol{l_4 sin(\theta_4)}\}$$. (Eq. 57).

Supplementary Note 3

Contributions of viscoelastic effects to epithelial tension

Viscous dissipations inside the polarized epithelium could provide significant contributions to the measured epithelial tension. On various tissues, there is not such a comprehensive study on viscoelasticity, thus we would like to investigate if viscous dissipation is considerable. Proceeding with the calculation of the ratio of viscoelastic contribution to epithelial tension is:

$$\frac{\boldsymbol{Viscosity}}{\boldsymbol{Tension}} = \frac{\boldsymbol{2}}{\boldsymbol{f}\frac{\boldsymbol{d\phi}}{\boldsymbol{df}}} \text{ . (Eq. 58).}$$

We can use Eqsu58 to estimate the viscoelastic contribution to epithelial tension of a polarized monolayer using the following parameters: f=38 kHz and $\frac{d\phi}{df}$=0.035 °/Hz. The obtained viscosity-tension ratio is 0.086, thus the viscoelastic correction is small <10%. Therefore, we can safely neglect viscoelastic correction to epithelial tension.

Supplementary References

1 Green, C. P. & Sader, J. E. Frequency response of cantilever beams immersed in viscous fluids near a solid surface with applications to the atomic force microscope. *Journal of Applied Physics* **98**, 114913 (2005).

2 Gavara, N. & Chadwick, R. S. Noncontact microrheology at acoustic frequencies using frequency-modulated atomic force microscopy. *Nat Meth* **7**, 650-654 (2010).

3 Chadwick, R. S. & Liao, Z. High-Frequency Oscillations of a Sphere in a Viscous Fluid near a Rigid Plane. *SIAM Review* **50**, 313-322 (2008).

4 Chadwick, R. S. & Cartagena-Rivera, A. X. Using noncontact AFM frequency shifts to determine stereocilia bundle stiffness and tension in the developing cochlear sensory epithelium. *AIP Conference Proceedings* **1703**, 030012 (2015).

5 Courant, R. & Hilbert, D. *Methods of mathematical physics*. Vol. 1 (New York: Interscience Publishers, 1953).
